# Brahma-related gene-1 promotes tubular senescence and renal fibrosis through Wnt/β-catenin/autophagy axis

**DOI:** 10.1042/CS20210447

**Published:** 2021-08-06

**Authors:** Wangqiu Gong, Congwei Luo, Fenfen Peng, Jing Xiao, Yiqun Zeng, Bohui Yin, Xiaowen Chen, Shuting Li, Xiaoyang He, Yanxia Liu, Huihui Cao, Jiangping Xu, Haibo Long

**Affiliations:** 1 Department of Nephrology, Zhujiang Hospital, Southern Medical University, Guangzhou 510280, China; 2 Department of Laboratory Medicine, Zhujiang Hospital, Southern Medical University, Guangzhou 510280, China; 3 Traditional Chinese Pharmacological Laboratory, Third Level Research Laboratory of State Administration of Traditional Chinese Medicine, School of Traditional Chinese Medicine, Southern Medical University, Guangzhou 510515, China; 4 School of Pharmaceutical Sciences, Southern Medical University, Guangzhou 510515, China

**Keywords:** autophagy, Brahma-related gene 1, cellular senescence, renal fibrosis, Wnt/β-catenin

## Abstract

Although accelerated cellular senescence is closely related to the progression of chronic kidney disease (CKD) and renal fibrosis, the underlying mechanisms remain largely unknown. Here, we reported that tubular aberrant expression of Brahma-related gene 1 (BRG1), an enzymatic subunit of the SWItch/Sucrose Non-Fermentable complex, is critically involved in tubular senescence and renal fibrosis. BRG1 was significantly up-regulated in the kidneys, predominantly in tubular epithelial cells, of both CKD patients and unilateral ureteral obstruction (UUO) mice. *In vivo*, shRNA-mediated knockdown of BRG1 significantly ameliorated renal fibrosis, improved tubular senescence, and inhibited UUO-induced activation of Wnt/β-catenin pathway. In mouse renal tubular epithelial cells (mTECs) and primary renal tubular cells, inhibition of BRG1 diminished transforming growth factor-β1 (TGF-β1)-induced cellular senescence and fibrotic responses. Correspondingly, ectopic expression of BRG1 in mTECs or normal kidneys increased p16^INK4a^, p19^ARF^, and p21 expression and senescence-associated β-galactosidase (SA-β-gal) activity, indicating accelerated tubular senescence. Additionally, BRG1-mediated pro-fibrotic responses were largely abolished by small interfering RNA (siRNA)-mediated p16^INK4a^ silencing *in vitro* or continuous senolytic treatment with ABT-263 *in vivo*. Moreover, BRG1 activated the Wnt/β-catenin pathway, which further inhibited autophagy. Pharmacologic inhibition of the Wnt/β-catenin pathway (ICG-001) or rapamycin (RAPA)-mediated activation of autophagy effectively blocked BRG1-induced tubular senescence and fibrotic responses, while bafilomycin A1 (Baf A1)-mediated inhibition of autophagy abolished the effects of ICG-001. Further, BRG1 altered the secretome of senescent tubular cells, which promoted proliferation and activation of fibroblasts. Taken together, our results indicate that BRG1 induces tubular senescence by inhibiting autophagy via the Wnt/β-catenin pathway, which ultimately contributes to the development of renal fibrosis.

## Introduction

Chronic kidney disease (CKD) is increasingly regarded as a global public health problem, as it affects approximately 10–15% adults worldwide [1]. Epidemiologic studies have confirmed the higher prevalence and mortality of CKD in the elderly population [2]. Clinically, irrespective of the underlying aetiology of CKD, elderly patients are at a higher risk for CKD progression [3]. With ageing, certain pathological changes are frequently observed in the kidneys, such as glomerulosclerosis, tubular atrophy and interstitial fibrosis [4,5]. These characteristic changes in ageing kidneys are similar to those in CKD, suggesting a close link between ageing process and CKD progression.

The mechanistic basis of kidney ageing is cellular senescence, which is an irreversible state of cell-cycle arrest accompanied by a series of changes in cell morphology and epigenetics, induced by various forms of stress [6]. Mechanically, senescence programme is mainly induced and maintained through activating ARF-p53-p21 and p16^INK4a^-retinoblastoma (Rb) pathways [7]. Despite growth arrest, senescent cells (SCs) remain metabolically active, secreting numerous cytokines to influence the surrounding microenvironment, which is referred to as the senescence-associated secretory phenotype (SASP) [8]. The classical SASP includes proinflammatory cytokines such as interleukin (IL)-1, IL-6, monocyte chemotactic protein-1 (MCP-1, CCL2), and pro-fibrotic mediators such as plasminogen activator inhibitor-1 (PAI-1), transforming growth factor-β1 (TGF-β1), and matrix metalloproteinases (MMPs) [9,10]. In addition to the aforementioned CDK inhibitors and SASP factors, the increased activity of senescence-associated β-galactosidase (SA-β-gal) is also commonly used to identify SCs [11].

Cellular senescence, identified by senescence markers SA-β-Gal, p16^INK4a^ and/or p21, has been reported to be accelerated in multiple experimental animal models and human kidney diseases, which correlates with renal dysfunction and disease progression [12–18]. During ageing and renal diseases, SCs can be detected in many anatomical sites of the kidneys, predominantly in tubular epithelial cells [19,20]. Tubular senescence hinders the recovery of injured cells, thus leading to maladaptive repair [21]. Furthermore, SCs accumulation may contribute to pro-fibrotic responses through the secretion of SASP factors [22]. Therefore, the accumulation of tubular SCs is closely related to the progression of renal fibrosis [12,13]. Recently, multiple studies have confirmed the important driving role of tubular senescence in renal fibrosis [23–25]. Thus, a better understanding of the underlying mechanism of tubular senescence is essential for the development of targeted therapeutics for renal fibrosis.

Brahma-related gene 1 (BRG1), encoded by the *SMARCA4* gene, is a core catalytic ATPase subunit of the SWItch/Sucrose Non-Fermentable chromatin remodelling complex, which performs fundamental roles in gene regulation and cell lineage specification by altering chromatin structure using energy from ATP hydrolysis [26,27]. Yang et al. uncovered that BRG1 interaction with CD44 endows mesenchymal progenitor cells with cell-autonomous fibrogenicity, and conveys them to fibroblastic focus in idiopathic pulmonary fibrosis (IPF), thus drives the progression of IPF [28]. The fibrogenic role of BRG1 in liver has also been proved by genetic ablation of BRG1 in hepatic progenitor cell, which strongly suppressed liver fibrosis [29,30]. In kidney disease, Naito et al. [31] found that BRG1 increased the transcription of TNF-α and MCP-1 in renal ischaemia, and Liu et al. [32] demonstrated that endothelial-specific deletion of BRG1 alleviates renal injury in unilateral ureteral obstruction (UUO) mice. But the role of BRG1 in renal fibrosis remains largely elusive.

BRG1 has been implicated in cell cycle regulation and ageing process of cancer cells in very different ways. Hendricks et al. [33] and Napolitano et al. [34] showed that BRG1 can cause cell cycle arrest and induce senescence of breast cancer cells and mesenchymal stem cells, respectively. Conversely, Wang et al. [35] demonstrated that BRG1 could bind to SIRT1, inactivate the p53/p21 pathway, suppress cellular senescence, and promote cell proliferation in colorectal cancer (CRC) cells. To date, the precise role and underlying mechanisms of BRG1 on senescence of normal diploid cells in kidney disease remain unclear .

Wnt/β-catenin pathway is an evolutionarily conserved pathway, which plays critical roles in various biological processes, such as embryonic development, tumorigenesis and tissue repair [36,37]. Aberrant activation of the Wnt/β-catenin pathway is associated with the development and progression of renal fibrosis and CKD [38–40]. Moreover, activation of the Wnt/β-catenin pathway plays a decisive role in driving tubular senescence during renal fibrosis [23]. Importantly, Baker et al. [41] found that β-catenin can recruit BRG1 to bind to its target gene promoter and activate transcription. Subsequent studies further confirmed this earlier finding showing that BRG1 could regulate various physiological and pathological processes via activation of the Wnt/β-catenin pathway [42–44]. However, there are few studies to explore their interaction and its possible mechanisms in kidney disease.

In the present study, we demonstrated that BRG1 was induced in the kidneys, specifically in tubular epithelial cells, of CKD patients and UUO mice. We revealed the potential role of BRG1 in tubular senescence and renal fibrosis *in vivo* and *in vitro* and further explored its underlying mechanisms. Our results establish a critical role for BRG1 in the pathogenesis of renal fibrosis.

## Materials and methods

### Animal models

Male C57BL/6J mice (weighing 20–23 g) were obtained from Southern Medical University Animal Center (Guangzhou, China). Mice were raised in a standard environment on a regular light/dark cycle with free access to chow and water. All animal experimental protocols were approved by the Southern Medical University Ethics Committee (Approval No. L2019050) and conducted in Southern Medical University.

The UUO mouse model was established by double-ligating the left ureter using a 4-0 silk after a midline abdominal incision under anaesthesia, as described previously [45]. For sham-operated mice, the ureters were exposed and manipulated but not ligated. To investigate the effects of BRG1, two sets of mouse experiments were conducted. The detailed experimental designs are shown in Figures 2A and 4A. *In vivo* knockdown or expression of BRG1 in mice was performed by a hydrodynamic-based gene delivery approach, as described previously [39,46]. Briefly, the mouse BRG1 shRNA sequences (5′-CCATCATGGAAGACTACTT-3′) were ligated on to an shRNA expression plasmid (pGPH1-shRNA) (GenePharma, Shanghai, China), and groups of mice were administered shRNA expression plasmid (pGPH1-shBRG1) or BRG1 expression plasmid (pReceiver-M14-BRG1) (GenePharma) by rapid injection of a large volume of DNA solution through the tail vein. In the first set of experiments, three groups of mice were used (*n*=6 per group): (1) sham control, (2) UUO mice injected with empty shRNA plasmid pGPH1-vector, and (3) UUO mice injected with pGPH1-shBRG1. Mice were killed by exsanguination under anaesthesia with inhaled 5% isoflurane in room air at day 10 after UUO. Kidneys were collected for further analysis. In the second set of experiments, after unilateral nephrectomy (UNx) under anaesthesia, the mice were divided into four groups as follows (*n*=6 per group): (1) mice injected with empty vector pReceiver-M14 and treated with vehicle (10% ethanol, 30% polyethylene glycol 400, and 60% Phosal 50 PG) (MCE, NJ, U.S.A.) by gavage, (2) mice injected with pReceiver-M14 vector and treated with ABT-263 (50 mg/kg per day for 5 days per cycle for two cycles, with a 1-week interval between cycles) (Selleck Chemicals, TX, U.S.A.) by gavage, as described previously [47], (3) mice injected with pReceiver-M14-BRG1 plasmid and treated with vehicle, and (4) mice injected with pReceiver-M14-BRG1 plasmid and treated with ABT-263. At 9 weeks after UNx, all mice were killed by exsanguination under anaesthesia with inhaled 5% isoflurane in room air and kidneys were collected for further analysis.

### Histology and immunohistochemical staining

The renal tissue was fixed in 4% paraformaldehyde, and paraffin-embedded kidney sections (4-μm-thickness) were prepared by a routine procedure. Masson’s trichrome staining was performed according to the manufacturer’s procedures (Leagene Biotechnology, Beijing, China). For each animal, ten randomly selected fields of Masson’s trichrome staining were observed under a light microscope (×400). To examine tubulointerstitial collagen deposition, the blue-stained area was semiquantitatively calculated as the fibrotic area using Image-Pro Plus 6.0 software (Media Cybernetics, MD, U.S.A.). Immunohistochemical staining was conducted as previously described [48]. The primary antibodies used in this study were as follows: anti-BRG1 (ab110641, Abcam, U.K.), anti-fibronectin (ab2413, Abcam), anti-collagen I (BA0325, BOSTER, China), anti-α-SMA (ab5694, Abcam), anti-p16^INK4a^ (ab189034, Abcam), and anti-TGFβ1 (sc-146, Santa Cruz Biotechnology, U.S.A.).

### Western blot analysis

Total protein sample from kidney tissues or cells were extracted with RIPA lysis buffer (Beyotime, Shanghai, China) on ice. The supernatants were then collected after centrifugation at 10000×***g*** at 4°C for 15 min. Cytoplasmic and nuclear proteins from mouse renal tubular epithelial cells (mTECs) were separated by a commercial protein separation kit (Thermo Fisher Scientific, MA, U.S.A.) according to the manufacturer’s procedures. The protein concentrations were quantified using the BCA Protein Assay Kit (Thermo Fisher Scientific, MA, U.S.A.). Protein expression was analysed by Western blot analysis as described previously [49]. The primary antibodies used were as follows: anti-BRG1 (ab110641, Abcam, U.K.), anti-fibronectin (ab2413, Abcam), anti-collagen I (BA0325, BOSTER, China), anti-α-SMA (ab5694, Abcam), anti-β-Actin (E021020, EarthOx Life Science, U.S.A.), anti-p16^INK4a^ (ab189034, Abcam), anti-p21 (ab109199, Abcam), anti-p19^ARF^ (ab202225, Abcam), anti-β-catenin (8480, Cell Signaling Technology, U.S.A.), anti-non-phospho (active) β-catenin (8814, Cell Signaling Technology) anti-MMP7 (3801, Cell Signaling Technology), anti-Snail-1 (3879, Cell Signaling Technology), anti-PAI-1 (sc-5297, Santa Cruz Biotechnology), anti-Histone H3 (4499, Cell Signaling Technology), anti-TGFβ1 (sc-146, Santa Cruz Biotechnology), anti-LC3A/B (12741, Cell Signaling Technology), anti-SQSTM1 (23214, Cell Signaling Technology), and anti-Beclin-1 (ab210498, Abcam).

### Cell culture and treatment

mTECs were provided by Dr Jeffrey B. Kopp (NIH, Bethesda, MD) and cultured as described previously [45]. Normal rat kidney interstitial fibroblast (NRK-49F) cells and HEK-293T cells were obtained from the American Type Culture Collection (ATCC, Manassas, VA, U.S.A.) and maintained according to the supplier’s protocol. For treatments, mTECs were stimulated with recombinant human TGF-β1 (5 ng/ml, R&D Systems, MN, U.S.A.), ICG-001 (5 µM, Selleck Chemicals, TX, U.S.A.), or CQ (20 μM, Sigma–Aldrich, Merck KGaA, Germany), as indicated.

For transfection, mTECs were transiently transfected with control vector (pReceiver-M14), BRG1 expression vector (pReceiver-M14-BRG1), BRG1-specific small interfering RNA (siRNA) or p16^INK4a^-specific siRNA (all from GenePharma, Shanghai, China) using the Lipofectamine 2000 reagent (Invitrogen, MA, U.S.A.) according to the manufacturer’s protocol, and HEK-293T cells were transfected with pReceiver-M14 or pReceiver-M14-BRG1. For some experiments, mTECs were transfected with BRG1-specific siRNA (or control siRNA) or pReceiver-M14-BRG1 (or pReceiver-M14), followed by incubation with TGF-β1 (5 ng/ml) or ICG-001 (5 µM), respectively. To assess autophagy, pReceiver-M14-BRG-tranfected mTECs were stimulated with ICG-001 (5 µM), followed by treatment with RAPA (200 nM, Selleck Chemicals) or Baf A1 (5 nM, Selleck Chemicals), as indicated. The Ctrl-CM and BRG1-CM were prepared by transfecting mTECs with pReceiver-M14 and pReceiver-M14-BRG1, respectively. NRK-49F cells were treated with Ctrl-CM and BRG1-CM in different proportions for the indicated time period.

Primary tubular epithelial cells were isolated from C57BL/6J mice according to procedures as previously described, with some modifications [50]. Briefly, mice were anaesthetised, and both kidneys were removed after cardiac perfusion. Subsequently, the kidneys were divided into tiny pieces and digested with 0.75 mg/ml collagenase I (Gibco, MA, USA) for 40 min at 37°C. Afterwards, the mashed samples were passed through sieves of descending pore sizes (100 and 40 μm, BD Biosciences, CA, U.S.A.). The tubular tissues were isolated using 31% Percoll gradients (Sigma–Aldrich), resuspended, and washed twice with PBS. The resultant tubular cell isolates were suspended and cultured in DMEM containing 10% foetal calf serum (Gibco), hormone mix (10 µg/ml EGF, 10 mM Hepes, 0.5 mg/ml prostaglandin E2, and 180 µg/ml hydrocortisone) (all from Sigma–Aldrich), 100 units/ml penicillin, and 100 µg/ml streptomycin (both from Gibco). Cells were cultivated in cell culture plates, and the medium was changed on day 3 and then every other day. Upon reaching 60–80% confluence, the cells were treated with TGF-β1 (5 ng/ml) or Etoposide (10 µM, Selleck Chemicals) and cotreated with or without BRG1 inhibitor PFI-3 (2 µM, Selleck Chemicals) in a serum-free medium for 7 days.

### Dual-luciferase reporter assay

mTECs and HEK-293T cells were co-transfected with TOPFlash TCF reporter plasmid, *Renilla* luciferase reporter plasmid (an internal control to normalise the transfection and harvest efficiencies) (both from GenePharma, Shanghai, China), and BRG1 expression vector (pReceiver-M14-BRG1) or control vector (pReceiver-M14) using Lipofectamine 2000 reagent. Cells were harvested 48 h after transfection, Firefly and *Renilla* luciferase activity were measured using a dual-luciferase reporter assay kit according to the manufacturer’s recommendations (Promega, CA, U.S.A.). The relative luciferase activity represents the ratio of the firefly luciferase activity to the *Renilla* luciferase activity.

### Immunofluorescence

mTECs or NRK-49F cells grown on coverslips were fixed with 4% formaldehyde and permeabilised with 1% Triton X-100. After blocking with 10% goat serum for 1 h, the cells were incubated with primary antibodies against E-cadherin (610181, BD Biosciences), β-catenin (8480, Cell Signaling Technology), or fibronectin (ab2413, Abcam). After washing, the cells were stained with DyLight 594-conjugated goat anti-rabbit or goat anti-mouse IgG (Abbkine, CA, U.S.A.), and the nuclei were counterstained with DAPI (BestBio, Shanghai, China). Images were captured by an inverted confocal microscope (ECLIPSE Ti, Nikon, Tokyo, Japan).

### RNA extraction and quantitative real-time PCR

Total RNA in mTECs was extracted using RNAiso reagent (Takara, Japan) and converted into cDNA using a Reverse Transcription System kit according to the manufacturer’s protocols (Takara). Quantitative real-time PCR was conducted using SYBR Green Premix (Takara) on the ABI PRISM 7500 Real-Time PCR System (Applied Biosystems, CA, U.S.A.) as previously described [48]. Primers used in the present study were synthesised by Sangon (Shanghai, China), and the primer sequences are listed in Supplementary Table S1.

### SA-β-gal staining

Frozen kidney sections (4 µm) or primary tubular epithelial cells were stained to detect SA-β-gal activity according to the manufacturer’s protocol (9860, Cell Signaling Technology).

### Cytokine array

To detect mTECs-secreted cytokines, the Mouse XL Cytokine Array Kit (ARY028, R&D Systems) was used following the manufacturer’s instructions. The signals were detected by chemiluminescence and measured by Image-Pro Plus 6.0 software (Media Cybernetics, MD, U.S.A.).

### Enzyme-linked immunosorbent assay

Mouse TGF-β1, IL-6, TNF-α, and CCL2 levels in the supernatants of mTECs were measured using enzyme-linked immunosorbent assay (ELISA) kits (EK0515, EK0411, EK0527, EK0568, BOSTER, China) in accordance with manufacturer’s instructions.

### Statistical analysis

All data are presented as the mean ± SD. Statistical analysis was performed with SPSS 20.0 (IBM, NY, U.S.A.). Student’s *t* test was used to analyse the differences between two groups, and one-way analysis of variance was used for comparisons across multiple groups, followed by either a Bonferroni’s *post hoc* test or Dunnett’s T3 test based on the results of a homogeneity of variance test. *P*<0.05 was considered to indicate statistical significance.

## Results

### BRG1 is up-regulated in multiple types of clinical nephropathy and experimental model of renal fibrosis

**Figure 1:**
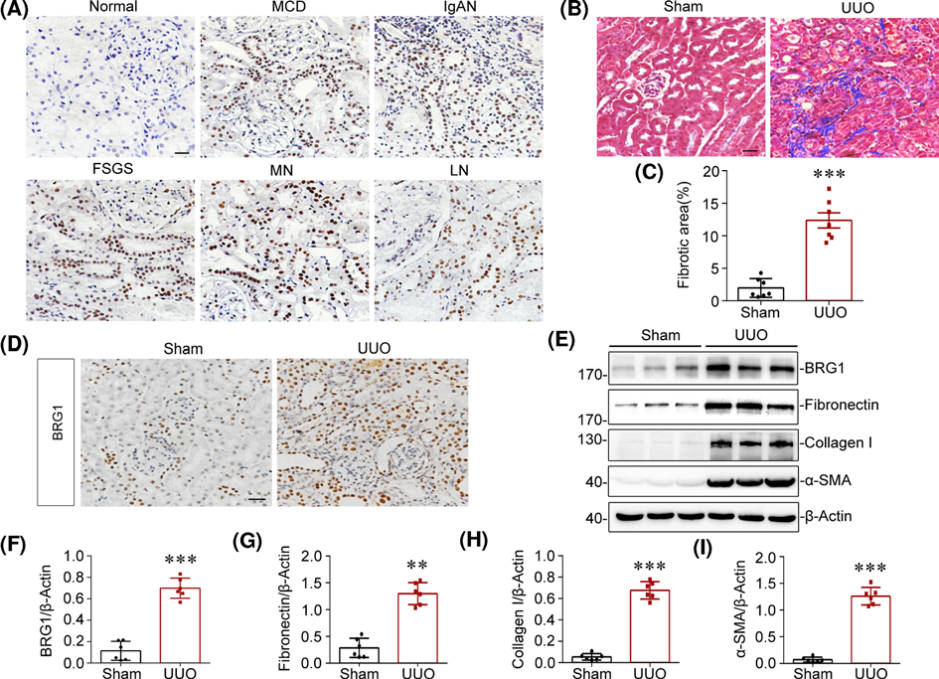
BRG1 is up-regulated in multiple types of clinical nephropathy and UUO model (**A**) Representative immunohistochemical micrographs demonstrated the expression and localisation of BRG1 protein in the kidney tissue of CKD patients. Non-tumour kidney tissue from patients with renal carcinoma who underwent nephrectomy served as the control. Scale bar, 50 μm. (**B**) Masson’s trichrome staining of sham and UUO kidneys at 7 days after UUO. Blue staining indicates fibrotic collagen deposition. Scale bar, 50 μm. (**C**) Quantitative analysis of renal fibrotic area in sham and UUO mice. ***P<0.001 versus sham group (*n*=7). (**D**) Immunohistochemical analysis demonstrated that BRG1 protein was mainly up-regulated in the tubular epithelial cells of UUO kidneys. Scale bar, 50 μm. (**E–I**) Western blot analysis showed renal expression of BRG1, fibronectin, collagen I, and α-SMA in sham and UUO mice. Representative Western blot (E) and quantitative data on the relative abundance of BRG1 (F), fibronectin (G), collagen I (H), and α-SMA (I) proteins in two groups are presented. **P<0.01, ***P<0.001 versus sham group (*n*=6).

In the present study, we first examined the expression and localisation of BRG1 in various types of nephropathies. To this end, we performed immunostaining in kidney biopsy specimens from CKD patients with minimal change disease (MCD), IgA nephropathy (IgAN), focal segmental glomerular sclerosis (FSGS), membranous nephropathy (MN), and lupus nephritis (LN). As shown in Figure 1A, BRG1 protein was markedly up-regulated in all biopsy specimens from patients with CKD, but was hardly detectable in normal control kidneys. Notably, BRG1 was predominantly distributed in the tubular epithelial cells in human diseased kidneys. To further determine the role of BRG1 in the pathogenesis of renal fibrosis, we examined BRG1 expression in a mouse CKD model induced by UUO. Compared with sham-operated mice, Masson’s trichrome staining showed a remarkable increase in collagen deposition in the obstructed kidneys of UUO mice (Figure 1B,C). Simultaneously, immunohistochemical staining demonstrated that BRG1 was markedly up-regulated in the UUO kidneys, which primarily distributed in renal tubular epithelium (Figure 1D). To quantitatively determine the relative abundance of BRG1 protein, western blot analysis of whole kidney lysates was performed. As shown in Figure 1E,F, compared with sham control, BRG1 expression was significantly elevated in the obstructed kidneys, accompanied by an increase in fibrosis markers, including fibronectin, collagen I, and α-smooth muscle actin (α-SMA) (Figure 1E,G–I). Collectively, these data implicated that BRG1 induction may be a common pathological feature in CKD of various aetiologies, suggesting a potential role of BRG1 in the development of renal fibrosis.

### *In vivo* knockdown of BRG1 ameliorates renal fibrosis after UUO

**Figure 2:**
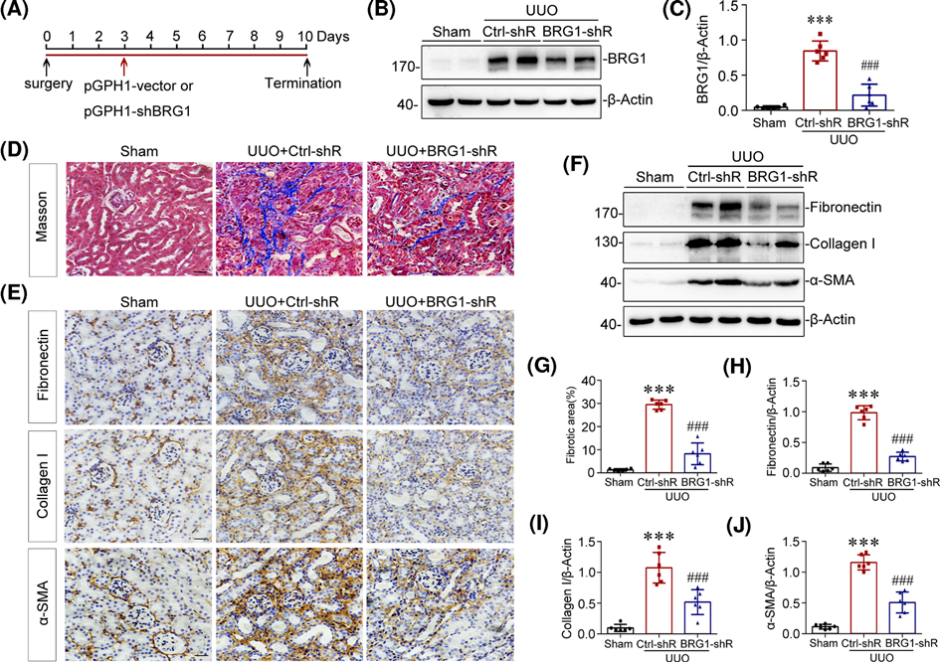
Knockdown of BRG1 ameliorates renal fibrosis in UUO mice (**A**) Experimental design. Red arrow indicates the injection of pGPH1-shBRG1 (BRG1-shR) or pGPH1-vector (Ctrl-shR) plasmids. Black arrows indicate the timing of UUO surgery. (**B,C**) Western blot analysis showed that renal expression of BRG1 was successfully knockdown after injection with BRG1-shRNA plasmids in UUO mice. Representative Western blot of BRG1 (B) and quantification results (C) are presented. ***P<0.001 versus sham group; ^###^*P*<0.001 versus UUO alone (*n*=6). (**D**) Masson’s trichrome staining revealed that tubulointerstitial collagen deposition were reduced by knockdown of BRG1 in UUO mice. Scale bar, 50 μm. (**E**) Immunohistochemical analysis demonstrated fibronectin, collagen I, and α-SMA protein expression in three groups as indicated. Scale bar, 50 μm. (**F**) Western blot analysis showed that renal fibronectin, collagen I, and α-SMA proteins expression were significantly inhibited by knockdown of BRG1 in UUO mice. (**G**) Graphic representation of renal fibrotic area in three groups after quantitative determination. (**H–J**) Graphic representations of renal fibronectin (H), collagen I (I), and α-SMA (J) proteins expression in three groups as indicated. ***P<0.001 versus sham group; ^###^*P*<0.001 versus UUO alone (*n*=6).

To ascertain the pro-fibrotic role of BRG1 in renal fibrosis, mice were intravenously injected with an shRNA vector encoding the interference sequence targeting BRG1 (pGPH1-shBRG1) through a hydrodynamic-based gene delivery approach [39,46] (Figure 2A). We observed that renal expression of BRG1 was successfully knockdown in UUO mice after intravenous injection of BRG1 shRNA plasmid (Figure 2B,C). As shown in Figure 2D,G, knockdown of BRG1 markedly attenuated tubulointerstitial collagen accumulation, as evidenced by Masson’s trichrome staining. Consistently, immunostaining analysis demonstrated that the deposition of major interstitial matrix proteins fibronectin and collagen I were significantly decreased by BRG1 knockdown in UUO kidneys (Figure 2E). In addition, the activation of interstitial myofibroblasts was also blocked after BRG1 knockdown, as illustrated by the reduced α-SMA expression (Figure 2E). To quantify the extent of renal fibrosis, we analysed the expression levels of fibrosis markers by Western blot analysis of whole kidney lysates. As shown in Figure 2F,H–J, renal expression of fibronectin, collagen I, and α-SMA in UUO mice were significantly attenuated after BRG1 knockdown. Together, these results indicated that BRG1 overexpression induced by UUO plays a critical role in promoting renal fibrosis.

### *In vivo* knockdown of BRG1 inhibits tubular senescence and activation of Wnt/β-catenin signalling pathway after UUO

**Figure 3:**
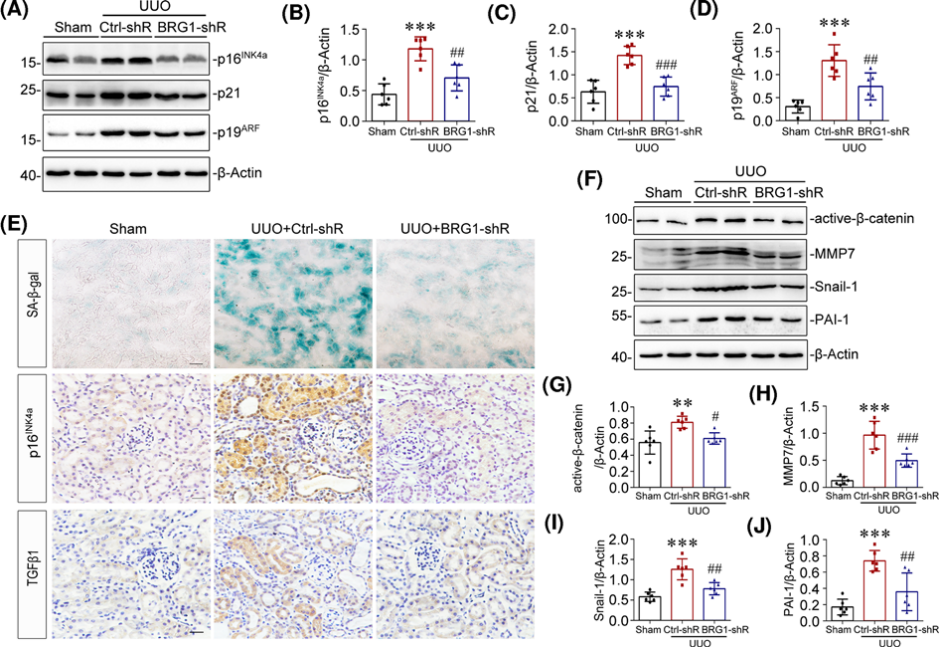
Knockdown of BRG1 inhibits tubular senescence and the activation of Wnt/β-catenin signalling pathway in UUO mice (**A–D**) Western blot analysis demonstrated that knockdown of BRG1 attenuated renal expression of p16^INK4a^, p21, and p19^ARF^ proteins induced by UUO. Representative Western blot (A) and quantification of protein levels of renal p16^INK4a^ (B), p21 (C), and p19^ARF^ (D) are presented. (**E**) Representative staining micrographs showed renal SA-β-gal activity, p16^INK4a^, and TGF-β1 expression in different groups as indicated. Frozen kidney sections were stained for SA-β-gal activity, paraffin-embedded kidney sections were immunostained with antibodies against p16^INK4a^ or TGF-β1. Scale bar, 50 μm. (**F–J**) Western blot analysis showed that renal expression of active-β-catenin, MMP-7, snail-1, and PAI-1 were significantly inhibited by knockdown of BRG1 in UUO mice. Representative western blot (F) and quantitative data (G–J) are presented. **P<0.01, ***P<0.001 versus sham group; ^#^*P*<0.05, ^##^*P*<0.01, ^###^*P*<0.001 versus UUO alone (*n*=6).

It has been recognised that accelerated cellular senescence contributes to the pathogenesis of renal fibrosis [23]. Since BRG1 has been implicated in cellular senescence of many cancer cells [33–35], we further investigated if BRG1 knockdown altered tubular senescence *in vivo*. As shown in Figure 3A–D, we found that knockdown of BRG1 largely inhibited the up-regulation of senescence-related signalling proteins p16^INK4a^, p21 and p19^ARF^ in the UUO kidneys. This observation was supported by immunohistochemical staining results, which showed that *in vivo* knockdown of BRG1 significantly suppressed UUO-induced SA-β-gal activity and p16^INK4a^ expression in tubular cells (Figure 3E). Further, tubule-derived TGF-β1 secretion was blocked after BRG1 knockdown (Figure 3E). These data suggested that knockdown of BRG1 inhibited UUO-induced tubular senescence, indicating a critical role of BRG1 in the acceleration of tubular senescence in renal fibrosis.

Additionally, when exploring the possible mechanisms by which BRG1 promotes tubular senescence in renal fibrosis, we detected the activation of Wnt/β-catenin signalling, which has previously been demonstrated as a driving factor in renal tubular senescence [23,51]. As shown in Figure 3F,G, Western blot analysis revealed that renal expression of non-phospho-β-catenin (active-β-catenin) was substantially inhibited by BRG1 knockdown in UUO mice. Moreover, knockdown of BRG1 also significantly attenuated renal expression of the key transcriptional targets of Wnt/β-catenin, including MMP-7, snail-1 and PAI-1, all of which are verified pathogenic mediators of renal fibrosis [52,53] (Figure 3F,H–J). These data demonstrated that BRG1 knockdown strikingly prevented the activation of Wnt/β-catenin pathway in the UUO kidney.

### Clearance of SCs inhibits BRG1-induced renal fibrosis in mice

**Figure 4:**
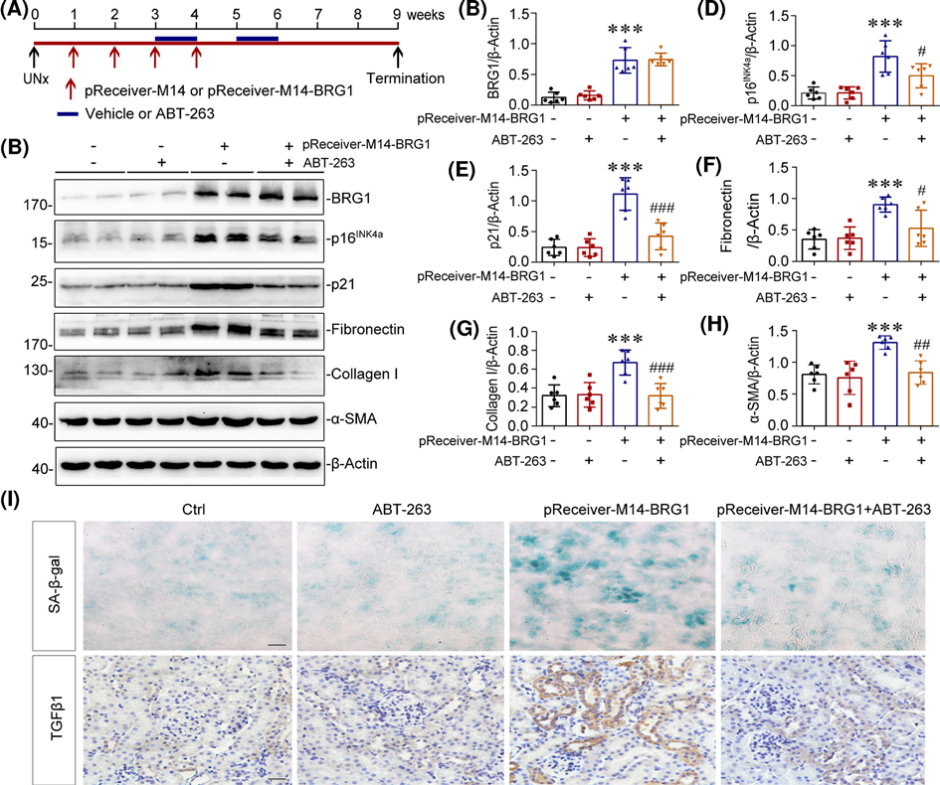
Clearance of SCs inhibits BRG1-induced renal fibrosis in mice (**A**) Experimental design. Red arrows indicate the injection of control (pReceiver-M14) or BRG1 overexpression plasmid (pReceiver-M14-BRG1). Blue lines indicate the administration of vehicle or ABT-263. (**B**) Representative western blot demonstrated the renal expression of BRG1, p16^INK4a^, p21, fibronectin, collagen I, and α-SMA proteins in different groups as indicated. (**C–H**) Quantitative data on the relative abundance of renal BRG1 (C), p16^INK4a^ (D), p21 (E), fibronectin (F), collagen I (G), and α-SMA (H) proteins in four groups as indicated. ***P<0.001 versus control group; ^#^*P*<0.05, ^##^*P*<0.01, ^###^*P*<0.001 versus pReceiver-M14-BRG1 injection alone (*n*=6). (**I**) Representative staining micrographs showed renal SA-β-gal activity and TGF-β1 expression in four groups as indicated. Frozen kidney sections were stained for SA-β-gal activity and paraffin-embedded kidney sections were immunostained with antibodies against TGF-β1. Scale bar, 50 μm.

To establish the causative link between BRG1-induced cellular senescence and renal fibrosis, mice undergoing UNx were subjected to BRG1 overexpression through intravenous injection of BRG1 expression vector (pReceiver-M14-BRG1) or empty vector (pReceiver-M14) for 4 weeks to induce tubular senescence and fibrotic response. Subsequently, these mice were treated with senolytic agent ABT-263 or vehicle for two cycles (Figure 4A). ABT-263 has been proven to be a highly selective senolytic agent, which selectively kills SCs through the inhibition of anti-apoptotic proteins BCL-2 and BCL-xL [54,55]. Efficient BRG1 overexpression in mice was shown by Western blot analysis of whole kidney lysates (Figure 4B,C). The accumulation of SCs in kidneys was confirmed by increases in p16^INK4a^ and p21 expression (Figure 4B,D,E) and up-regulation of SA-β-gal activity (Figure 4I) after BRG1 overexpression. In addition, increased expression of BCL-xL and BCL-2 proteins were detected in the BRG1 overexpression group (Supplementary Figure S1), which is consistent with previous studies that SCs are resistant to apoptosis in part owing to up-regulation of Bcl-xL and/or BCL-2 [55,56]. Simultaneous inhibition of BCL-2 and BCL-xL activity by ABT-263 (Supplementary Figure S1) effectively cleared renal accumulation of SCs induced by BRG1 overexpression, as evidenced by the reduced SA-β-gal activity (Figure 4I) and decreased p16^INK4a^ and p21 protein expression in the kidneys of the ABT-263-treated group (Figure 4B,D,E). Notably, renal expression of fibrotic markers induced by BRG1 overexpression, including fibronectin, collagen I, and α-SMA, were all markedly inhibited after ABT-263 treatment (Figure 4B,F–H). In addition, as shown in Figure 4I, immunostaining results showed that ABT-263 treatment significantly blocked the BRG1-induced SA-β-gal activity and TGF-β1 expression in tubules. As mentioned above, TGF-β1 is a vital component of SASP and has been recognised as the master modulator of fibrogenesis. Taken together, these data indicated that BRG1 overexpression promoted the development of renal fibrosis via inducing tubular senescence.

### BRG1 promotes fibrotic responses in renal tubular epithelial cells

**Figure 5:**
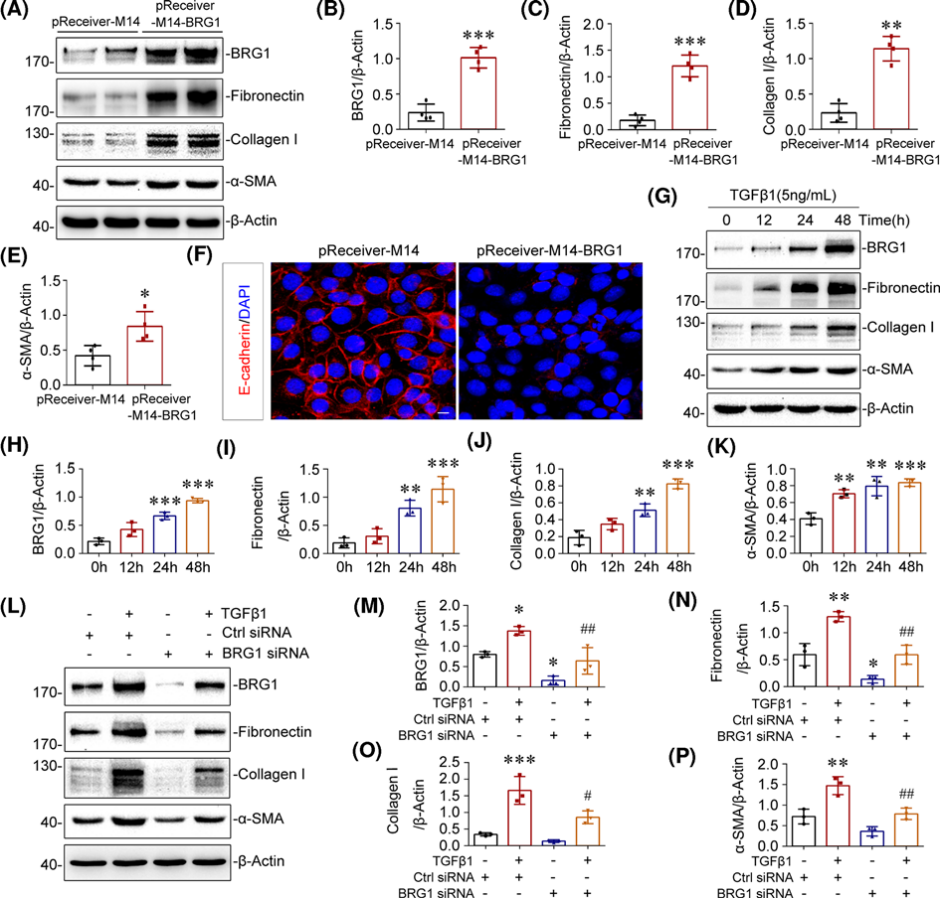
BRG1 promotes fibrotic responses in renal tubular epithelial cells (**A–E**) Western blot analysis showed that overexpression of BRG1 induced the protein levels of fibronectin, collagen I, and α-SMA *in vitro*. mTECs were transfected with control vector (pReceiver-M14) or BRG1 overexpression plasmid (pReceiver-M14-BRG1) for 48 h. Representative Western blot (A) and quantitative data on BRG1 (B), fibronectin (C), collagen I (D), and α-SMA (E) are presented. *P<0.05, **P<0.01, ***P<0.001 versus pReceiver-M14 group (*n*=4). (**F**) Representative immunofluorescent micrographs of E-cadherin (red) merged with DAPI (blue) in mTECs after transfection with pReceiver-M14 or pReceiver-M14-BRG1 for 48 h. Scale bar, 10 μm. (**G–K**) Western blot analysis showed that TGF-β1 time-dependently induced the expression of BRG1, fibronectin, collagen I, and α-SMA *in vitro*. mTECs were treated with TGF-β1 (5 ng/ml) for the indicated time period. Representative Western blot (G) and quantitative data on the relative abundance of BRG1 (H), fibronectin (I), collagen I (J), and α-SMA (K) proteins in four groups are presented. **P<0.01, ***P<0.001 versus control groups (*n*=3). (**L–P**) Western blot analysis showed that knockdown of BRG1 blocked TGF-β1-induced fibronectin, collagen I, and α-SMA proteins expression. mTECs were transfected with control (Ctrl siRNA) or BRG1-specific siRNA (BRG1 siRNA), followed by stimulation with TGF-β1 (5 ng/ml) for 48 h. Representative Western blot (L) and quantitative data on the relative abundance of BRG1 (M), fibronectin (N), collagen I (O), and α-SMA (P) proteins in different groups are presented. *P<0.05, **P<0.01, ***P<0.001 versus control group; ^#^*P*<0.05, ^##^*P*<0.05 versus Ctrl siRNA in the presence of TGF-β1 (*n*=3).

We further investigated the role of BRG1 on the fibrotic response in renal tubular epithelial cells. To this end, mTECs were transiently transfected with BRG1 expression vector (pReceiver-M14-BRG1) or empty vector (pReceiver-M14), and the overexpression efficacy was confirmed by western blot (Figure 5A,B). The occurrence of epithelial–mesenchymal transition (EMT) and extracellular matrix (ECM) secretion were induced in mTECs after BRG1 overexpression, as characterised by up-regulation of α-SMA and matrix proteins such as fibronectin and collagen I (Figure 5A,C–E) and down-regulation of E-cadherin [57] (Figure 5F), suggesting a pro-fibrotic role of BRG1 in tubular cells.

Because TGF-β1 is identified as a core regulator of fibrosis [58], we then explored whether BRG1 expression was regulated by TGF-β1 *in vitro*. Results of Western blot analysis showed that TGF-β1 substantially increased the expression of ECM proteins fibronectin, collagen I, and myofibroblast marker α-SMA in mTECs, which indicates the activation of EMT [57]. Notably, TGF-β1 significantly induced BRG1 protein expression in a time-dependent manner (Figure 5G–K). Given that TGF-β1 induced BRG1 expression, we next examined whether BRG1 is required for the pro-fibrotic effect of TGF-β1 in renal epithelial cells. Hence, we knocked down BRG1 expression using an siRNA strategy. mTECs were transfected with control (Ctrl siRNA) or BRG1-specific siRNA (BRG1 siRNA), followed by stimulation with TGF-β1. Efficient BRG1 knockdown was confirmed in whole-cell lysates by Western blot analysis (Figure 5L,M). As shown in Figure 5L–P, exogenous TGF-β1 markedly induced BRG1 expression. By contrast, BRG1 interference significantly blocked TGF-β1-induced production of fibronectin, collagen I, and α-SMA in mTECs. These results demonstrated that interference of BRG1 prevented the pro-fibrotic response induced by TGF-β1 in tubular epithelial cells. Considering the master role of TGF-β1 in fibrotic response, these data strongly indicated that BRG1 would be a crucial determinant of renal fibrosis downstream of TGF-β1.

### BRG1 induces cellular senescence in renal tubular epithelial cells

**Figure 6:**
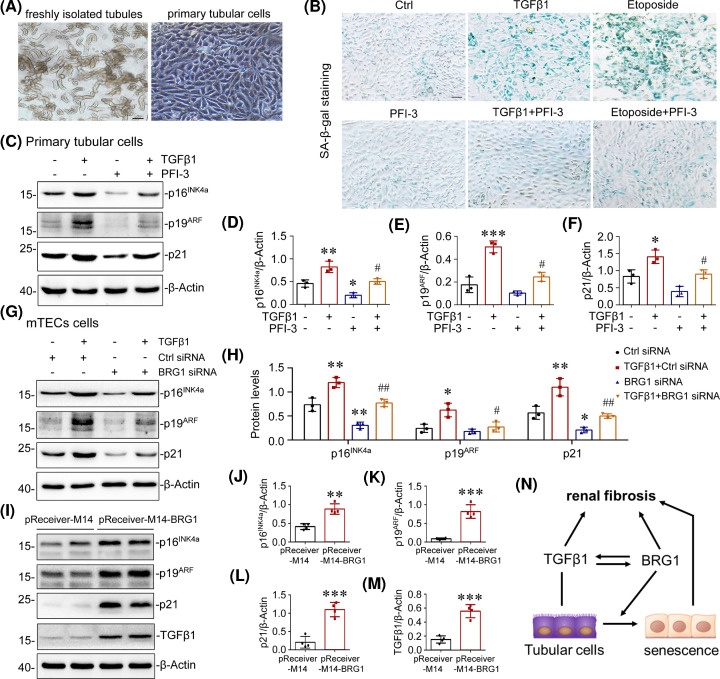
BRG1 induces cellular senescence in renal tubular epithelial cells (**A**) Representative micrographs showed freshly isolated tubules and cultured primary tubular epithelial cells. Scale bar, 100 μm. (**B**) Representative micrographs showed staining for SA-β-gal activity of primary tubular cells in different groups as indicated. The primary tubular cells were treated with TGF-β1 (5 ng/ml) or etoposide (10 µM) in the absence or presence of BRG1 inhibitor PFI-3 (2 μM) for 7 days before staining. Scale bar, 100 μm. (**C–F**) Western blot analysis demonstrated that pharmacologic inhibition of BRG1 blocked TGF-β1-induced p16^INK4a^, p19^ARF^, and p21 proteins expression in primary tubular cells. The primary tubular cells were incubated with TGF-β1 (5 ng/ml) in the absence or presence of BRG1 inhibitor PFI-3 (2 µM) for 7 days. Representative Western blot (C) and quantitative data on the relative abundance of p16^INK4a^ (D), p19^ARF^ (E), and p21 (F) proteins in four groups are presented. *P<0.05, **P<0.01 versus control group; ^#^*P*<0.05 versus TGF-β1 alone (*n*=3). (**G–H**) Western blot analysis showed that knockdown of BRG1 inhibited TGFβ1-induced p16^INK4a^, p19^ARF^, and p21 protein expression in tubular epithelial cell line. mTECs were transfected with control (Ctrl siRNA) or BRG1-specific siRNA (BRG1 siRNA), followed by stimulation with TGF-β1 (5 ng/ml) for 48 h. Representative Western blot (G) and quantitative data on the relative abundance of p16^INK4a^, p19^ARF^, and p21 proteins in different groups as indicated (H) are presented. *P<0.05, **P<0.01 versus control group; ^#^*P*<0.05, ^##^*P*<0.01 versus Ctrl siRNA in the presence of TGF-β1 (*n*=3). (**I–M**) Western blot analysis showed that overexpression of BRG1 induced the expression of p16^INK4a^, p19^ARF^, p21, and TGF-β1 in tubular epithelial cell line. mTECs were transfected with control vector (pReceiver-M14) or BRG1 overexpression plasmid (pReceiver-M14-BRG1) for 48 h. Representative Western blot (I) and quantitative data on the relative abundance of p16^INK4a^ (J), p19^ARF^ (K), p21 (L), and TGF-β1 (M) proteins are presented. **P<0.01, ***P<0.001 versus pReceiver-M14 group (*n*=4). (**N**) Schematic representation of interaction between BRG1 and TGF-β1 forms a reciprocal activation loop, jointly promoting tubular senescence and renal fibrosis. TGF-β1 induces the expression of BRG1, and knockdown of BRG1 blocked TGFβ1-induced tubular senescence and renal fibrosis. Similarly, in pathologic conditions, BRG1 up-regulation induces tubular senescence and renal fibrosis, BRG1 up-regulation also increases the expression of TGF-β1, which further promotes tubular senescence and renal fibrosis.

We then examined the role of BRG1 in cellular senescence *in vitro*. Renal tubules were isolated from mouse kidneys and cultivated for use as primary tubular cells (Figure 6A). Subsequently, we incubated primary tubular cells with TGF-β1 [59] or etoposide (a classical senescence inducer, used as a positive control) [60] to induce premature senescence, with or without the pre-treatment of BRG1 inhibitor PFI-3. PFI-3 is a potent and cytoactive small-molecule inhibitor that functions through selective binding to bromodomain of BRG1/Brahma-associated factors (BAFs) and blocks the interaction of BRG1 with acetylated histone, mimicking the effect of BRG1 deletion [61]. As shown in Figure 6B, blockage of BRG1 suppressed the SA-β-gal activity induced by TGF-β1 or etoposide in primary tubular cells. Consistently, Western blot analysis showed that TGFβ1-induced overexpression of p16^INK4a^, p19^ARF^, and p21 were largely blocked by pre-treatment of PFI-3 in primary tubular cells (Figure 6C–F). Similar results were obtained when BRG1 was knockdown by BRG1 siRNA in cultured tubular cell line mTECs (Figure 6G,H). In addition, overexpression of BRG1 significantly induced the expression of p16^INK4a^, p19^ARF^, p21, and TGF-β1 in mTECs (Figure 6I–M). These data demonstrated that BRG1 plays a critical role in mediating cellular senescence in tubular epithelial cells. Furthermore, the interaction between BRG1 and TGF-β1 formed a positive feedback loop, jointly promoting tubular senescence and renal fibrosis (Figure 6N).

To further clarify the causality between BRG1-induced pro-fibrotic responses and tubular senescence, mTECs were co-transfected with BRG1 expression vector (pReceiver-M14-BRG1) and p16^INK4a^-specific siRNA. The transfection efficiency was confirmed by Western blot, as shown by the nearly abolished BRG1-induced expression of p16^INK4a^ by p16^INK4a^ knockdown (Supplementary Figure S2A–C). As shown in Supplementary Figure S2A,D–F, knockdown of p16^INK4a^ significantly reduced the expression of fibronectin, collagen I, and α-SMA induced by BRG1 overexpression. Taken together, these findings revealed that BRG1 promotes the fibrotic response possibly through activation of the p16^INK4a^ signalling pathway in tubular cells.

### BRG1 mediates tubular senescence and fibrotic responses via Wnt/β-catenin pathway

**Figure 7:**
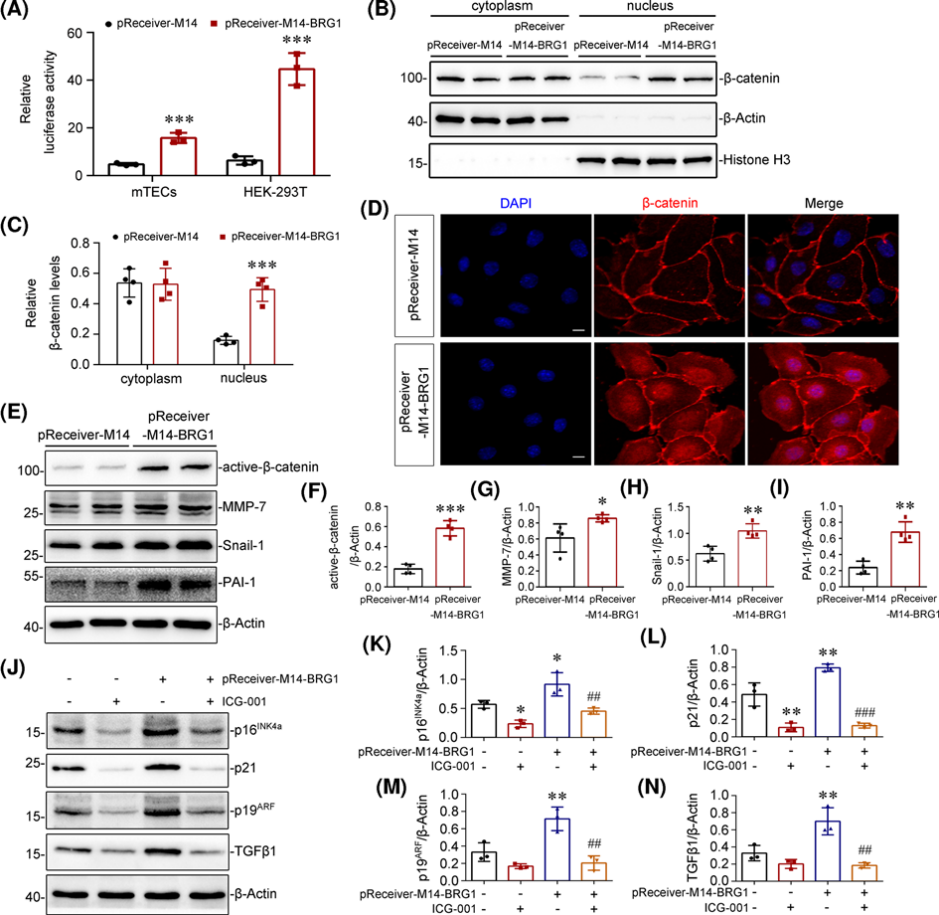
BRG1 mediates tubular senescence and fibrotic responses via Wnt/β-catenin pathway (**A**) BRG1 overexpression enhanced the Wnt/β-catenin-mediated gene transcription activity. mTECs and HEK-293T cells were co-transfected with TOPFlash reporter plasmid, *Renilla* luciferase reporter vector (an internal control to normalise the transfection efficiency) and BRG1 expression vector (pReceiver-M14-BRG1) or control vector (pReceiver-M14) for 48 h as indicated. ***P<0.001 versus pReceiver-M14 group (*n*=3). (**B,C**) Cytoplasmic and nuclear proteins in mTECs were isolated and Western blot analysis was performed to verify the nuclear translocation of β-catenin. mTECs were transfected with BRG1 expression vector (pReceiver-M14-BRG1) or control vector (pReceiver-M14) for 48 h. Representative Western blot (B) and quantitative data on the relative abundance of cytoplasmic and nuclear β-catenin protein (C) in two groups are presented. β-Actin and Histone-H3 were used as internal controls for the cytosolic or nuclear fraction, respectively. ***P<0.001 versus pReceiver-M14 group (*n*=4). (**D**) Immunofluorescence staining demonstrated that the expression and nuclear translocation of β-catenin were induced by BRG1 overexpression in mTECs. Scale bar, 10 μm. (**E–I**) Western blot analysis showed that overexpression of BRG1 increased the expression of active-β-catenin, MMP-7, snail-1, and PAI-1. In mTECs, representative Western blot (E) and quantitative data on the relative abundance of active-β-catenin (F), MMP-7 (G), snail-1 (H) and PAI-1 (I) proteins in two groups are presented. *P<0.05, **P<0.01 versus pReceiver-M14 group (*n*=4). (**J–N**) Western blots analysis showed that BRG1-induced p16^INK4a^, p21, p19^ARF^, and TGF-β1 expression were blocked by pharmacologic inhibition of Wnt/β-catenin pathway *in vitro*. mTECs were transfected with pReceiver-M14-BRG1, followed by stimulation with Wnt/β-catenin signalling inhibitor ICG-001 (5 µM) for 48 h. Representative Western blot (J) and quantitative data on the relative abundance of p16^INK4a^ (K), p21 (L), p19^ARF^, (M) and TGF-β1 (N) proteins are presented. *P<0.05,**P<0.01 versus control group; ^##^*P*<0.01, ^###^*P*<0.001 versus pReceiver-M14-BRG1 alone (*n*=3).

We further explored whether the activation of Wnt/β-catenin signalling is required for BRG1 in mediating tubular senescence and fibrotic response in tubular epithelial cells. We first performed a TOPFlash/*Renilla* luciferase reporter experiment to evaluate the effect of BRG1 on the Wnt/β-catenin-mediated transcription activity *in vitro*. As shown in Figure 7A, relative luciferase activity was significantly enhanced by transfection of BRG1 overexpression vector (pReceiver-M14-BRG1) in both mTECs and HEK-293T cells, as expected. Since the accumulation of β-catenin in the nucleus is an essential step of Wnt/β-catenin pathway activation [62]. We next explore the distribution of β-catenin in cytoplasm and nucleus. The Western blot results showed that BRG1 overexpression significantly increased the nuclear localisation of β-catenin in mTECs (Figure 7B,C). And the nuclear translocation of β-catenin induced by BRG1 overexpression was further validated by immunofluorescence (Figure 7D). We also analysed the protein level of active-β-catenin and several key target genes of Wnt/β-catenin signalling, including MMP7, snail-1, and PAI-1. Western blot results showed that the expression of these proteins was significantly elevated after BRG1 overexpression in mTECs (Figure 7E–I). These data demonstrated that overexpression of BRG1 activates the Wnt/β-catenin pathway *in vitro.* Since activation of the Wnt/β-catenin pathway requires binding of Wnt ligands and Wnt receptors, we next examined the effect of BRG1 overexpression on the expression of Wnt ligands and Wnt receptors by real-time PCR. We found that BRG1 overexpression increased the expression levels of various Wnt ligands (including Wnt1, Wnt2b, Wnt3, WNT3a, Wnt6, Wnt7a, Wnt8b, Wnt9a, and Wnt16) and several Wnt receptors (including FZD4, FZD5, and LRP5), but had no effect on Axin1, DVL1/2, and GSK3β mRNA levels (Supplementary Figure S3).

To further detect the role of Wnt/β-catenin pathway in BRG1-induced tubular senescence and fibrotic responses, we used a pharmacologic approach to inhibit Wnt/β-catenin pathway by treating mTECs with a small molecule inhibitor, ICG-001 [63]. As shown in Figure 7J–N, ICG-001 treatment suppressed BRG1-induced expression of p16^INK4a^, p21, p19^ARF^, and TGF-β1 in mTECs. Further, the expression of fibrotic markers up-regulated by BRG1 overexpression, including fibronectin, collagen I, PAI-1 and α-SMA, were reversed after ICG-001 intervention (Supplementary Figure S4). Together, these results suggested that BRG1 mediates tubular senescence and fibrotic responses dependent on the activation of Wnt/β-catenin signalling pathway.

### BRG1-dependent Wnt/β-catenin pathway activation promotes tubular senescence through autophagic inhibition

**Figure 8:**
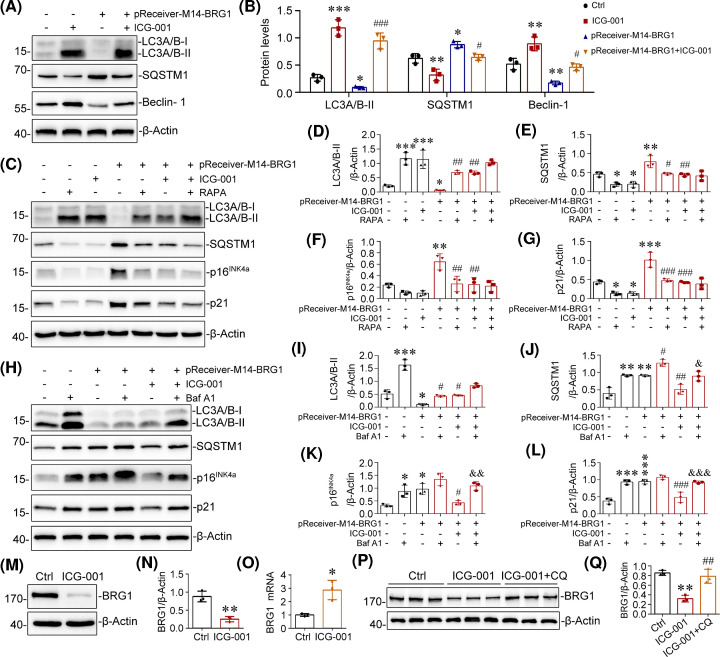
BRG1-dependent Wnt/β-catenin pathway activation promotes tubular senescence through autophagic inhibition (**A,B**) Western blot analysis demonstrated that BRG1-mediated autophagy inhibition was reversed by pharmacologic inhibition of Wnt/β-catenin signalling. mTECs were transfected with BRG1 expression vector (pReceiver-M14-BRG1), followed by stimulation with ICG-001 (5 µM) for 48 h. Representative Western blot (A) and quantitative data on the relative abundance of LC3A/B-II, SQSTM1, and Beclin-1 proteins in different groups (B) are presented. *P<0.05, **P<0.01, ***P<0.001 versus control group; ^#^*P*<0.05, ^###^*P*<0.001 versus pReceiver-M14-BRG1 transfection alone (*n*=3). (**C–G**) Western blots analysis showed that the activation of autophagy by RAPA blocked BRG1-induced p16^INK4a^ and p21 proteins expression. mTECs were transfected with pReceiver-M14-BRG1, followed by treatment with or without ICG-001 (5 µM, 48 h) and RAPA (200 nM, last 2 h) as indicated. Representative Western blot (C) and quantitative data on LC3A/B-II (D), SQSTM1 (E), p16^INK4a^ (F), and p21 (G) are presented. *P<0.05,**P<0.01, ***P<0.001 versus control group; ^#^*P*<0.05, ^##^*P*<0.01, ^###^*P*<0.001 versus pReceiver-M14-BRG1 transfection alone (*n*=3). (**H–L**) Western blot analysis showed that autophagy inhibition by Baf A1 eliminated the inhibition of BRG1-induced p16^INK4a^ and p21 protein expression by ICG-001. mTECs were transfected with pReceiver-M14-BRG1, followed by treatment with or without ICG-001 (5 µM, 48 h) and Baf A1 (5 nM, last 2 h) as indicated. Representative Western blot (H) and quantitative data on LC3A/B-II (I), SQSTM1 (J) p16^INK4a^ (K), and p21 (L) are presented. *P<0.05,**P<0.01, ***P<0.001 versus control group; ^#^*P*<0.05, ^##^*P*<0.01, ^###^*P*<0.001 versus pReceiver-M14-BRG1 transfection alone. ^&^*P*<0.05,^ &&^*P*<0.01, ^&&&^*P*<0.001 versus pReceiver-M14-BRG1 in the presence of ICG-001 (*n*=3). (**M,N**) Western blot analysis demonstrated that ICG-001 decreased the level of BRG1 protein expression *in vitro*. mTECs were stimulated with ICG-001 (5 µM) for 24 h. Representative Western blot of BRG1 (M) and quantitative data (**N**) are presented. **P<0.01 versus control groups (*n*=3). (**O**) Quantitative real-time polymerase chain reaction (qRT-PCR) showed that ICG-001 increased the mRNA level of BRG1 *in vitro*. mTECs were stimulated with ICG-001 (5 µM) for 24 h. *P<0.05 versus control groups (*n*=3). (**P,Q**) Western blot analysis showed that CQ recovered ICG-001-induced BRG1 protein down-regulation. mTECs were incubated with ICG-001 in the absence or presence of autophagy–lysosomal inhibitor CQ (20 μM) for 24 h. Representative Western blot of BRG1 (P) and quantitative data (Q) are presented. **P<0.01 versus control group; ^##^*P*<0.01 versus ICG-001 alone (*n*=3). Abbreviations: Baf A1, bafilomycin A1; CQ, chloroquine; RAPA, rapamycin.

The above findings indicated that BRG1 promotes tubular senescence through the activation of Wnt/β-catenin signalling. Next, we investigated the underlying mechanism by which BRG1/Wnt/β-catenin modulates tubular senescence. As accumulating evidence has proposed the association between autophagy deficiency and accelerated senescence [64,65], we asked whether autophagy is required for BRG1/Wnt/β-catenin-induced tubular senescence. Our Western blot results showed that BRG1 overexpression inhibited the activation of autophagy, as shown by decreased expression of autophagy biomarkers LC3-II and Beclin-1 [66] and increased expression of cargo protein SQSTM1/p62 in mTECs. However, ICG-001, the specific inhibitor of Wnt/β-catenin signalling, reversed these alterations (Figure 8A,B). These results indicated that BRG1 inhibited autophagy via activating Wnt/β-catenin signalling.

Given that BRG1/Wnt/β-catenin could inhibit autophagy, we further examined the involvement of autophagy in BRG1/Wnt/β-catenin-induced tubular senescence. We first used rapamycin (RAPA, an mTOR inhibitor) as an inducer of autophagy. RAPA treatment markedly increased the expression of LC3-II and decreased SQSTM1 levels (Figure 8C–E), suggesting that autophagy was effectively activated. Notably, RAPA hindered BRG1-induced tubular senescence to a similar extent as ICG-001 treatment, as evidenced by reduced p16^INK4a^ and p21 expression (Figure 8C,F,G). In contrast, bafilomycin A1 (Baf A1) treatment, a late-stage autophagic flux inhibitor, significantly induced tubular senescence to a similar extent as BRG1 overexpression since it significantly up-regulated p16^INK4a^ and p21 levels, and Baf A1-mediated autophagy blockade was confirmed by SQSTM1 accumulation. Moreover, Baf A1 could reverse the inhibiting effect of ICG-001 on BRG1-induced tubular senescence (Figure 8H–L). Thus, these data implied that the inhibition of autophagy by BRG1/Wnt/β-catenin is required for promoting tubular senescence.

Interestingly, we observed that the inhibition of Wnt/β-catenin signalling by ICG-001 significantly decreased the protein level of BRG1 but increased its mRNA level (Figure 8M–O), indicating that ICG-001 may increase BRG1 protein degradation. Because ICG-001 was observed to profoundly activate autophagy, we sought to determine whether ICG-001 mediated BRG1 protein degradation through the autophagy–lysosomal pathway (ALP), one of the major protein degradation pathways [67,68]. To this end, mTECs were treated with chloroquine (CQ) to inhibit ALP-induced protein degradation in the presence of ICG-001. As shown in Figure 8P,Q, inhibiting lysosomal acidification and autophagy by CQ recovered ICG-001-induced BRG1 protein down-regulation in mTECs, indicating that ALP likely participated in ICG-001-induced BRG1 protein degradation.

### Tubular-SASP induced by BRG1 facilitates the activation of fibroblasts

**Figure 9:**
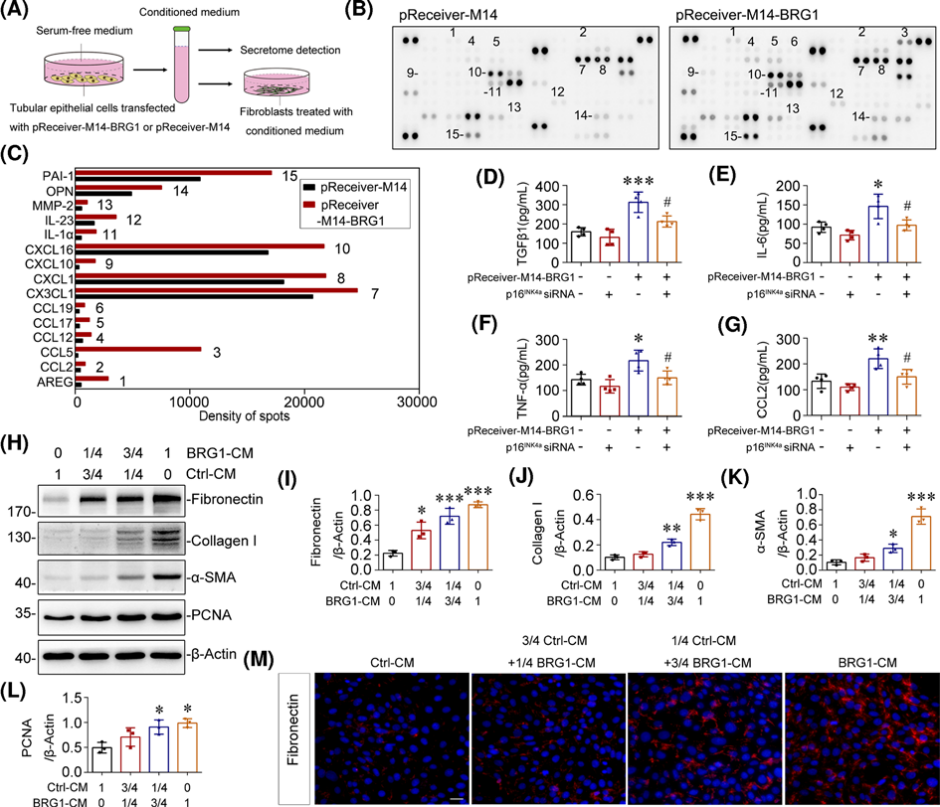
Tubular-SASP induced by BRG1 facilitates the activation of fibroblasts (**A**) Experimental procedures. mTECs were transfected with BRG1 overexpression plasmid (pReceiver-M14-BRG1) to induce cellular senescence or with empty vector (pReceiver-M14) as controls. The supernatants were collected for secretome detection or used for conditioned medium to stimulate normal rat kidney interstitial fibroblast (NRK-49F) cells (Ctrl-CM or BRG1-CM). (**B**) mTECs were transfected with pReceiver-M14 (Left) or pReceiver-M14-BRG1 (Right) for 48 h, then the supernatants were collected and subjected to cytokine array. (**C**) Spots were subjected to densitometry using Image J. Graphic presentations of changes of optical density in two groups as indicated. (**D–G**) ELISA results demonstrated the secretion of TGF-β1 (D), IL-6 (E), TNF-α (F), and CCL2 (G) of mTECs in different groups as indicated. mTECs were co-transfected with BRG1 expression plasmid (pReceiver-M14-BRG1) and p16^INK4a^-specific siRNA for 48 h, and then the supernatants were collected for ELISA detection. *P<0.05, **P<0.01, ***P<0.001 versus control group; ^##^*P*<0.01 versus pReceiver-M14-BRG1 transfection alone (*n*=4). (**H–L**) Western blot analysis showed that BRG1-CM induced fibronectin, collagen I, α-SMA, and PCNA proteins expression in cultured fibroblasts. NRK-49F cells were stimulated with Ctrl-CM and BRG1-CM in the indicated proportion for 48 h. Representative Western blot (H) and quantitative data on the relative abundance of fibronectin (I), collagen I (J), α-SMA (K), and PCNA (L) proteins in different groups are presented. *P<0.05, **P<0.01, ***P<0.001 versus control group (*n*=3). (**M**) Representative immunofluorescent micrographs of fibronectin (red) merged with DAPI (blue) in NRK-49F cells after stimulation with Ctrl-CM and BRG1-CM in the indicated proportion for 48 h. Scale bar, 50 μm.

Among many other phenotypic changes, premature senescence is well recognised to be accompanied by permanent transition of the secretome, also known as SASP [69]. Through secreting SASP factors, SCs exhibit potent paracrine activities on neighbouring cells and tissues in a continuous manner [9,10]. To further confirm the role of BRG1 in communication between tubular cells and interstitial fibroblasts, we transfected mTECs with the BRG1 expression vector (pReceiver-M14-BRG1) or empty vector (pReceiver-M14) and collected the supernatant as conditioned medium (BRG1-CM or Ctrl-CM) for secretome detection and fibroblasts treatment (Figure 9A). As detected by proteome array, BRG1 overexpression enhanced the secretion of multiple SASP components in mTECs, such as IL-1α, MMP-2, PAI-1, and various chemokines (Figure 9B,C). Further, ELISA revealed a significant increase in several classical SASP factors, including TGF-β1, IL-6, TNFα and CCL2, in the BRG1 overexpression group, and these effects were nearly eliminated by p16^INK4a^ knockdown (Figure 9D–G), suggesting an important role of p16^INK4a^-Rb pathway in BRG1-induced SASP formation. Treatment of fibroblasts (NRK-49F) with BRG1-CM concentration-dependently induced the secretion of fibronectin and collagen I, as detected by Western blot analysis and immunofluorescence (Figure 9H–J,M). Simultaneously, BRG1-CM enhanced the expression of PCNA and α-SMA, suggestive of the proliferation of fibroblasts and fibroblast-to-myofibroblast transdifferentiation (Figure 9H,K–L). Together, these results suggested that overexpression of BRG1 drives the secretion of SASP factors in tubular cells, leading to tubulo–interstitial cross-talk and, eventually, promoting myofibroblast activation and the progression of renal fibrosis.

## Discussion

Renal fibrosis is the major pathologic process driving progression of CKD. Recently, growing evidences have suggested that renal tubular cells are possible initiators of renal fibrosis [70]. However, the mechanism through which tubular cells induce renal fibrosis is poorly understood. In the present study, we identified chromatin remodelling protein BRG1, which was significantly up-regulated in renal tubular epithelial cells in multiple kidney diseases and UUO model, as an important pro-fibrotic mediator in renal fibrosis. More importantly, data presented in the present study confirmed the pro-fibrotic role of BRG1 is closely related to the induction of tubular senescence. Mechanistically, our study demonstrated that BRG1 promotes tubular senescence by inhibiting autophagy via the activation of Wnt/β-catenin pathway, which ultimately accelerates the progression of renal fibrosis.

BRG1, as a chromatin remodelling factor, directly disrupts the contact between histone and DNA to alter nucleosome structure and/or positioning, in an ATP-dependent manner, resulting in an open and accessible chromatin conformation conducive for essential factors required for transcription binding and thereby modulates the gene transcription [71,72]. Previous studies have reported the involvement of BRG1 in kidney diseases. For example, Naito et al*.* [31] suggested that BRG1 increases the transcription of TNF-α and MCP-1 in renal ischemia. However, still little evidence of BRG1 expression and its role in renal fibrosis is available. Here, we observed a remarkable elevation of BRG1 protein in the kidneys from both clinical CKD patient samples and the UUO model, indicating that BRG1 may be implicated in renal fibrosis. Despite previous studies demonstrating the effects of endothelial-derived BRG1 on the renal injury and fibrosis induced by ischemia-reperfusion injury [73] and UUO [32], our *in vivo* data have demonstrated that the expression of BRG1 was not confined to endothelial cells, especially in diseased human kidney tissues, BRG1 up-regulation was mainly observed in tubular epithelial cells (Figure 1). Thus, our study focused on the role of aberrant BRG1 in tubular epithelial cells in the context of renal fibrosis.

To ascertain the inherent effect of BRG1 in the development of renal fibrosis, we used two *in vivo* models, knockdown of BRG1 in UUO mice and maintenance overexpression of BRG1 in UNx mice, respectively. As the results shown by multiple detection methods, knockdown of BRG1 significantly prevented the progression of UUO-induced renal fibrosis (Figure 2), more impressively, overexpression of BRG1 without any other interventions after UNx activated fibrotic response directly, as demonstrated by the up-regulated expression of fibronectin, collagen I, and α-SMA in the original healthy kidney (Figure 4). Consistent with the *in vivo* observation, overexpression of BRG1 in tubular epithelial cells, was sufficient to induce fibrotic biomarkers corresponding to EMT and ECM accumulation (Figure 5). These observations unambiguously provide strong evidence for BRG1 in promoting renal fibrosis.

During these *in vivo* experiments, what caught our attention is the alteration of senescent status of renal tubular cells, which is always associated with the progression of renal fibrosis [6,74].Emerging evidences have proposed that the accelerated senescence of tubular cells serves as a driving force in the development of renal fibrosis [21,23–25,75]. Meanwhile, some studies have shown that BRG1 is involved in the regulation of senescence of various cancer cells. However, the specific role of BRG1 in cellular senescence appears to be controversial [76–79]. Tu et al. [80] demonstrated that BRG1 anchored to the promotors of p16^INK4a^-and p21- encoding genes was enhanced during senescence and was associated with the up-regulated expression of p16^INK4a^ and p21 in a chromatin remodelling activity-dependent manner. However, contradictory results have been obtained by Wang et al. [35] showing that BRG1 inhibited p53 expression by promoting SIRT1-mediated deacetylation of p53 at K382 via binding interaction in CRC cells. To date, the specific role of aberrant BRG1 expression in renal tubular senescence has not yet been elucidated. To clarify this issue, we isolated and cultivated primary tubular epithelial cells for use. These cells were treat with TGF-β1 or etoposide to induce premature senescence, and some of them were pre-treated with BRG1 inhibitor PFI-3. As the results shown by SA-β-gal staining and senescence protein markers detection, BRG1 inhibition significantly delayed cellular senescence of primary tubular cells. Correspondingly, ectopic expression of BRG1 in mTECs activated senescence-associated signalling pathways, as shown by the up-regulated levels of p16^INK4a^, p21 and p19^ARF^(Figure 6). More convincing data come from *in vivo*, maintenance overexpression of BRG1 independently accelerates tubular senescence in original healthy mouse kidney, which means that the persistent up-regulation of BRG1 is sufficient to induce premature senescence of tubular cells in renal fibrosis (Figure 4).

Our data also established a strong causative link of BRG1-induced tubular senescence and renal fibrosis. We found that BRG1-mediated pro-fibrotic responses were largely abolished by continuous senolytic treatment with ABT-263 *in vivo* (Figure 4) or siRNA-mediated p16^INK4a^ silencing *in vitro* (Supplementary Figure S2). Simultaneously, the secretion of various pro-inflammatory cytokines induced by BRG1 was also inhibited by the knockdown of p16^INK4a^, which further illustrated that BRG1-mediated transition of tubular secretome phenotype is closely related to cellular senescence (Figure 9). Collectively, these data indicated that BRG1 exerted fibrotic action mainly via inducing tubular senescence, suggesting that targeting blockage of BRG1 may be a potential therapeutic strategy to improve cellular senescence and renal fibrosis in CKD.

TGF-β1 has been identified as a key regulator of fibrosis [58] as well as an ageing promoter in some cell types [81–83]. In this study, we found that BRG1 could be induced by TGF-β1 stimulation (Figure 5) and either treatment with BRG1 inhibitor PFI-3 [84,85] or siRNA-mediated BRG1 knockdown blocked TGFβ1-mediated cellular senescence in tubular epithelial cells (Figure 6), suggesting that BRG1 may act as a positive regulator of tubular senescence downstream of TGF-β1. Interestingly, our further *in vivo* and *in vitro* studies showed that TGF-β1 expression could be up-regulated by BRG1 overexpression (Figures 4 and 6). Taken together, these results suggested that the interaction between BRG1 and TGF-β1 forms a reciprocal activation loop, jointly promoting tubular senescence and renal fibrosis. However, further investigations are needed to clarify the specific mechanism of mutual regulation between TGF-β1 and BRG1.

Previous studies have implicated that Wnt/β-catenin signalling pathway could be activated by BRG1 in some biological systems [42–44]. Indeed, we confirmed that BRG1 plays a role in positive regulation of Wnt/β-catenin pathway activation. Our *in vivo* data demonstrated that shRNA-mediated knockdown of BRG1 efficiently inhibited the Wnt/β-catenin signalling activation induced by UUO (Figure 3). In addition, BRG1-induced Wnt/β-catenin activation *in vitro* was evidenced by the enhanced relative luciferase activity of TOPFlash reporter, the increased nuclear accumulation of β-catenin, as well as the up-regulation of active-β-catenin and several classical target genes of Wnt/β-catenin, including MMP7, snail-1, and PAI-1 (Figure 7). Further, we confirmed that BRG1 activated Wnt/β-catenin pathway through transcriptional activation of multiple Wnt ligands (Wnt1, Wnt2b, Wnt3, Wnt3a, Wnt6, Wnt7a, Wnt8b, Wnt9a, and Wnt16) and Wnt receptors (FZD4, FZD5, and LRP5) (Supplementary Figure S3). Notably, pharmacologic inhibition of Wnt/β-catenin by ICG-001 *in vitro* diminished BRG1-induced tubular senescence (Figure 7) and fibrotic responses (Supplementary Figure S4). Overall, these findings strongly suggested that BRG1-mediated tubular senescence and renal fibrosis could be, at least partly, attributed to activation of the Wnt/β-catenin pathway.

We also revealed the mechanism underlying BRG1/Wnt/β-catenin axis in inducing tubular senescence could be related to the inhibition of autophagy. Previous studies have provided evidence that Wnt/β-catenin signalling acts as a crucial negative regulator of autophagy activation [86–88]. In line with these findings, we observed that the inhibition of Wnt/β-catenin pathway by ICG-001 reversed BRG1-mediated autophagy suppression, suggesting that BRG1 inhibited autophagy through activating Wnt/β-catenin signalling. The functional relationship between autophagy and cellular senescence is complex. Several studies have shown that activation of autophagy promotes senescence [89–91], whereas other reports suggest that autophagy prevents senescence [92–96]. In this study, we found that activation of autophagy by either RAPA or ICG-001 treatment blocked the BRG1-induced tubular senescence, while in the presence of the autophagy inhibitor Baf A1, tubular senescence was further induced, suggesting that autophagy acts as an antisenescent role in BRG1/Wnt/β-catenin-induced tubular senescence. Furthermore, upon using Baf A1 to inhibit autophagy, we found that the inhibiting effect of ICG-001 on BRG1-induced tubular senescence was almost eliminated. Overall, these data indicated that the inhibition of autophagy by BRG1/Wnt/β-catenin axis is required for promoting senescence in mTECs.

ALP, one of the major protein degradation pathways, has a critical role in maintaining cellular homoeostasis [97]. We demonstrated in this study that inhibition of Wnt/β-catenin signalling by ICG-001 down-regulated BRG1 protein degradation through ALP approach (Figure 8), suggesting that BRG1-mediated Wnt/β-catenin signalling activation may inhibit autolysosomal degradation of BRG1 itself, which further up-regulates BRG1’s protein level in mTECs. The discovery of this mechanism constitutes another positive feedback loop in BRG1/Wnt/β-catenin/autophagy axis in addition to the interaction between BRG1 and TGF-β1.

Another important finding in the present study is that BRG1 promotes the communication between senescent tubular cells and interstitial fibroblasts. As shown by our results, overexpression of BRG1 significantly changed the secretion profile of tubular cells to a pro-inflammatory and pro-fibrotic secretome phenotype, which further promoted proliferation and transdifferentiation of fibroblasts (Figure 9). This finding explains how renal tubule-derived BRG1 promotes renal fibrosis from the perspective of traditional fibrosis formation mechanism. Notably, BRG1-induced SASP factors such as TGF-β1, IL-6, TNF-α, and CCL2 were almost abolished after knockdown of p16^INK4a^, suggesting a p16^INK4a^-dependent role of BRG1 in SASP induction in tubular senescence.

In summary, our data demonstrated that tubular aberrant expression of BRG1 is a common pathological feature of CKD and critically involved in tubular senescence through the inhibition of autophagy via activating Wnt/β-catenin pathway, which further promotes fibrotic responses through a vicious positive feedback with TGF-β1. BRG1 also induces fibroblast activation indirectly through tubule-derived SASP factors. Although further studies are warranted, these findings undoubtedly provide a new determinant pathological factor and potential target for the prevention of renal fibrosis.

## Clinical perspectives

- The potential role of BRG1 in tubular senescence and renal fibrosis *in vivo* and *in vitro* and its underlying mechanisms were explored.
- Aberrant up-regulation of BRG1, a common pathological feature of CKD, inhibits tubular autophagy through the activation of Wnt/β-catenin pathway, which accelerates tubular senescence and ultimately promotes the development of renal fibrosis.
- Our findings establish a critical role for BRG1 in the pathogenesis of renal fibrosis and provide a new determinant pathological factor and potential target for the prevention of renal fibrosis.

## Supplementary Material

Supplementary Figures S1-S6 and Table S1

## Data Availability

The data used to support the findings of the present study are available from the corresponding authors upon request.

## Abbreviations

α-SMA: α-smooth muscle actin
ALP: autophagy–lysosome pathway
Baf A1: bafilomycin A1
BRG1: Brahma-related gene 1
CKD: chronic kidney disease
CQ: chloroquine
ECM: extracellular matrix
ELISA: enzyme-linked immunosorbent assay
EMT: epithelial–mesenchymal transition
IL: interleukin
IPF: idiopathic pulmonary fibrosis
MCP-1: monocyte chemotactic protein-1
MMP: matrix metalloproteinase
mTEC: mouse renal tubular epithelial cell
PAI-1: plasminogen activator inhibitor-1
RAPA: rapamycin
RB: retinoblastoma
SASP: senescence-associated secretory phenotype
SA-β-gal: senescence-associated β-galactosidase
SC: senescent cell
siRNA: small interfering RNA
TGF-β1: transforming growth factor-β1
UNx: unilateral nephrectomy
UUO: unilateral ureteral obstruction
