# Supplementary material for: Brahma-related gene-1 promotes tubular senescence and renal fibrosis through Wnt/β-catenin/autophagy axis
Source: Clin Sci (Lond). 2021 Aug 6;135(15):1873–95. doi: 10.1042/CS20210447 (PMC8358963; doi:10.1042/CS20210447)
Supplement: Supplementary Figures S1-S6 and Table S1 [file CS-2021-0447_supp.pdf]

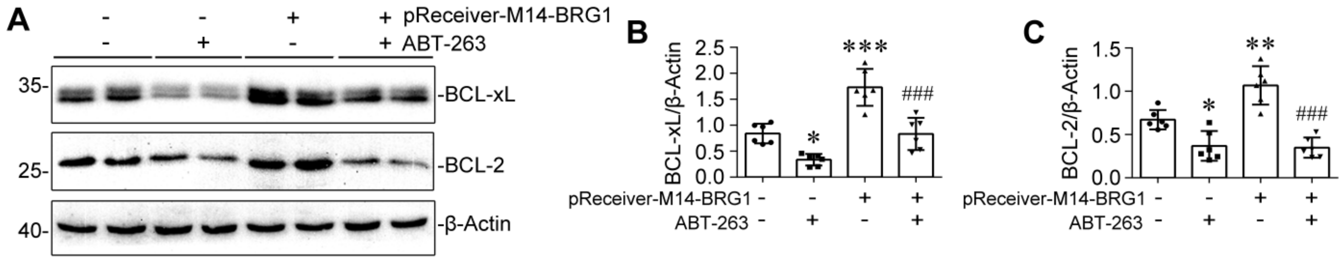

Supplementary Figure 1. The effects of ABT-263 treatment and BRG1 overexpression on the expression of BCL-xL and BCL-2 *in vivo*. (A-C) Western blot analysis showed renal expression of BCL-xL and BCL-2 proteins in four groups as indicated. Representative western blot (A) and quantitative data on the relative abundance of BCL-xL (B) and BCL-2 (C) proteins in different groups are presented. $^{*}P < 0.05$, $^{**}P < 0.01$, $^{***}P < 0.001$ versus control group; $^{###}P < 0.001$ versus pReceiver-M14-BRG1 injection alone (n=6).

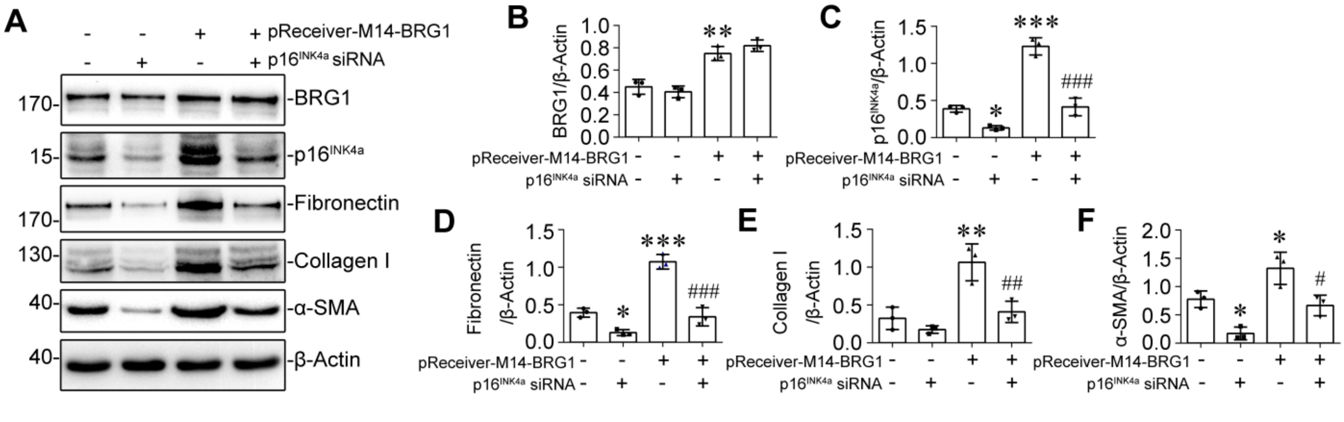

Supplementary Figure 2. Knockdown of $p16^{INK4a}$ inhibits BRG1-induced fibrotic responses in renal tubular epithelial cells. (A-F) Western blot analysis demonstrated that knockdown of $p16_{INK4a}$ inhibited BRG1-induced fibronectin, collagen I and α-SMA protein expression in cultured tubular epithelial cells. mTECs cells were co-transfected with BRG1 expression plasmid (pReceiver-M14-BRG1) and $p16^{INK4a}$-specific siRNA for 48 h. Representative western blot (A) and quantitative data on the relative abundance of BRG1 (B), $p16^{INK4a}$ (C), fibronectin (D), collagen I (E) and α-SMA (F) are presented. $^{*}P<0.05$, $^{**}P<0.01$, $^{***}P<0.001$ versus control group; $^{\#}P<0.05$, $^{\#\#}P<0.01$, $^{\#\#\#}P<0.001$ versus pReceiver-M14-BRG1 transfection alone (n=3).

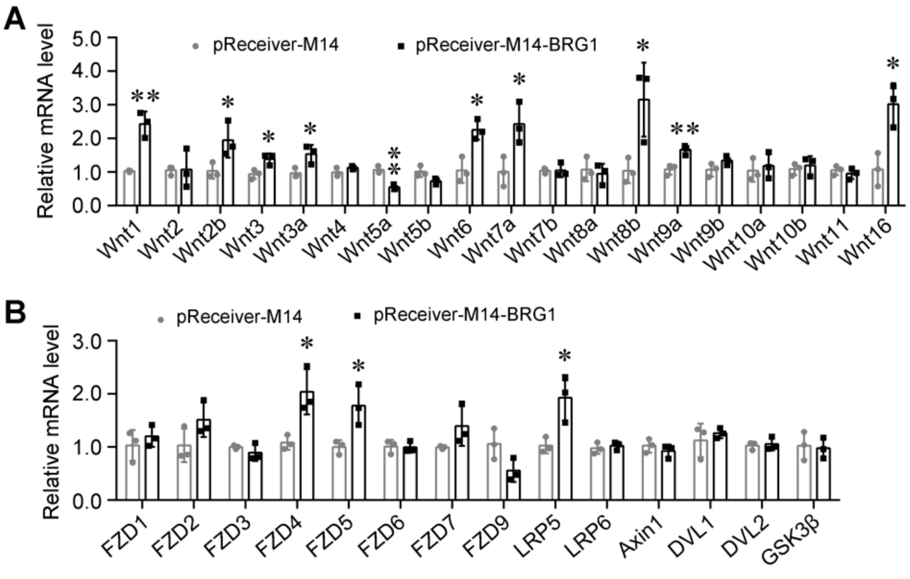

Supplementary Figure 3. The effects of BRG1 overexpression on the expression of Wnt ligands and Wnt receptors in renal tubular epithelial cells. (A-B) qRT-PCR results demonstrated the relative mRNA expression levels of Wnt ligands (A) and Wnt receptors (B) in mTECs cells in different groups as indicated. mTECs cells were transfected with control vector (pReceiver-M14) or BRG1 overexpression plasmid (pReceiver-M14-BRG1) for 48 h. $^{*}P < 0.05$, $^{**}P < 0.01$ versus pReceiver-M14 group (n=3).

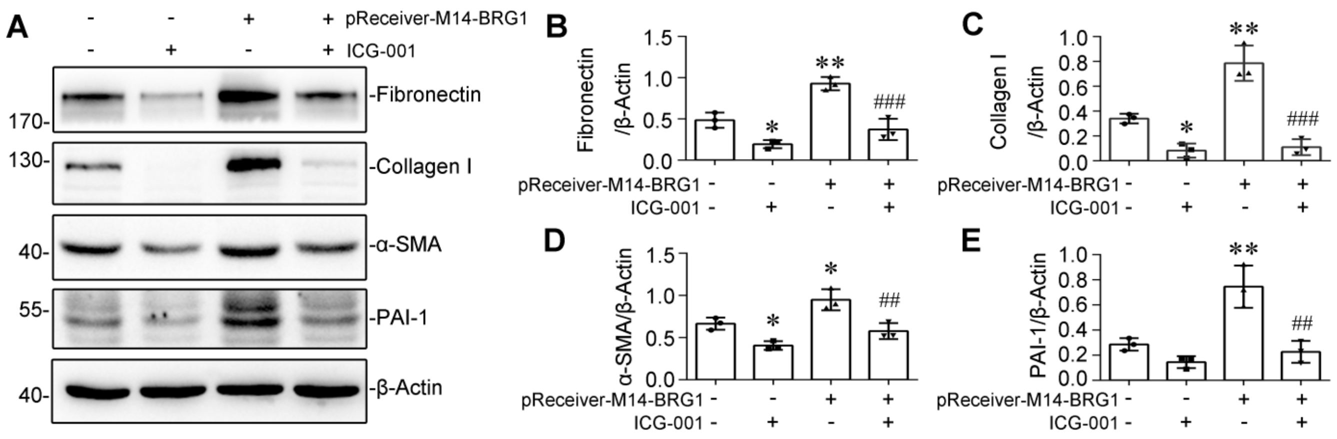

Supplementary Figure 4. ICG-001 inhibits BRG1-induced fibrotic responses in renal tubular epithelial cells. (A-E) Western blot analysis showed that BRG1-induced fibronectin, collagen I, α-SMA and PAI-1 expression were inhibited by ICG-001 treatment. mTECs cells were transfected with BRG1 expression vector (pReceiver-M14-BRG1), followed by stimulation with Wnt/β-catenin signalling inhibitor ICG-001 (5μM) for 48 h. Representative western blot (A) and quantitative data on the relative abundance of fibronectin (B), collagen I (C), α-SMA (D) and PAI-1 (E) proteins in different groups are presented. $^{*}P<0.05$, $^{**}P<0.01$ versus control groups; $^{\#\#}P<0.01$, $^{\#\#\#}P<0.001$ versus pReceiver-M14-BRG1 transfection alone (n=3).

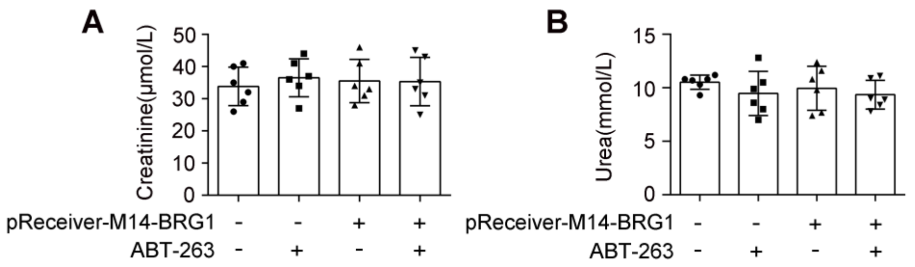

Supplementary Figure 5. Effects of ABT-263 treatment and BRG1 overexpression on the renal function in uninephrectomized mice. Renal function was evaluated by measuring serum creatinine (A) and serum urea (B) levels (n=6).

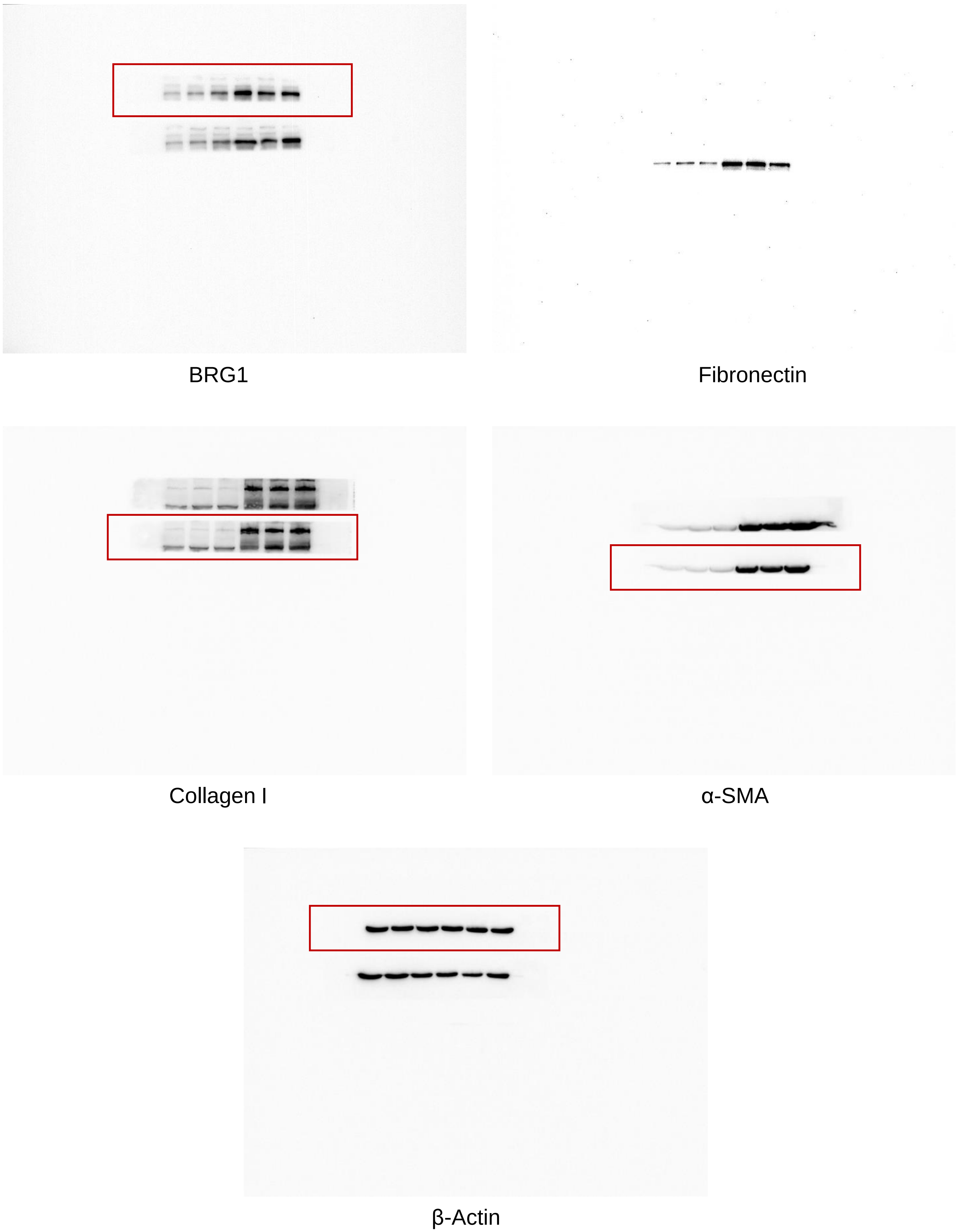

Figure 1

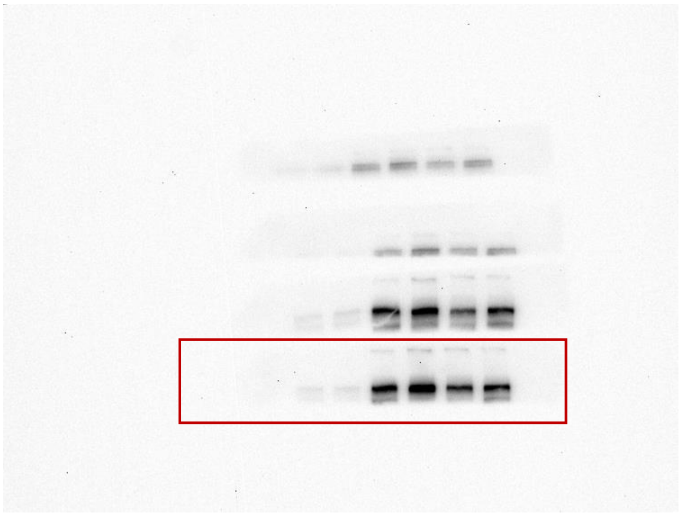

Figure 2B. BRG1

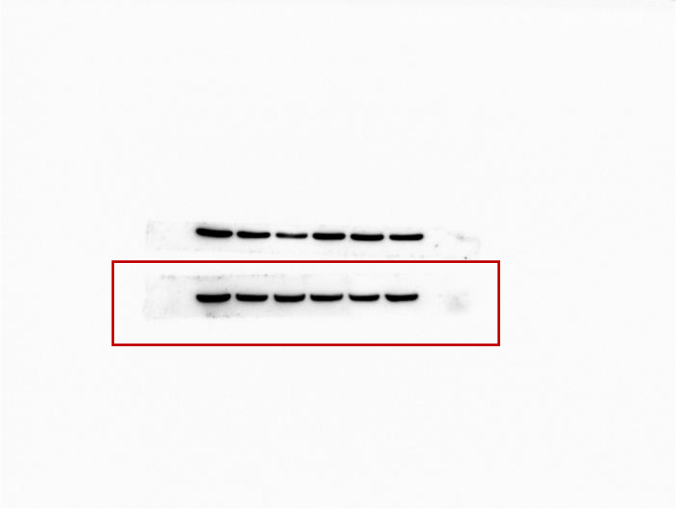

Figure 2B. β-Actin

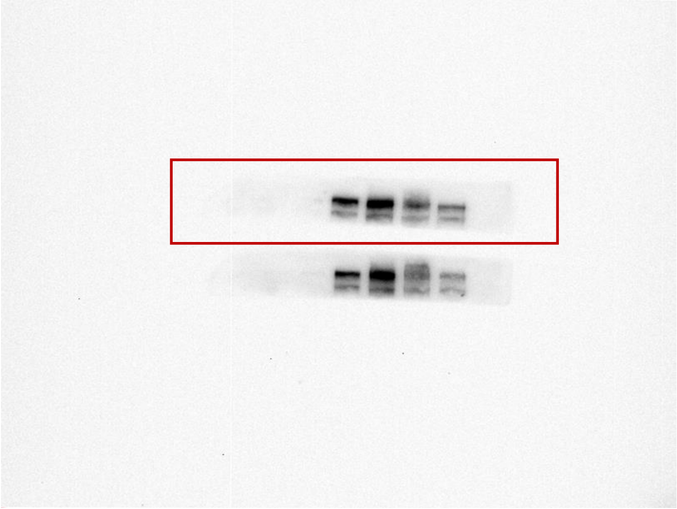

Figure 2F. Fibronectin

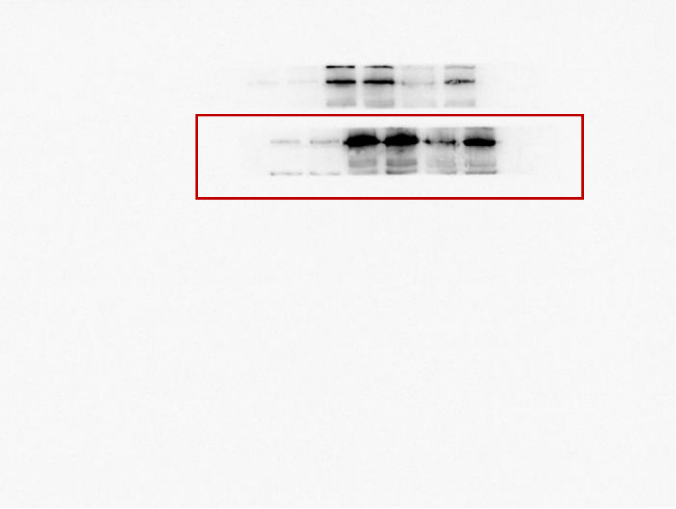

Figure 2F. Collagen I

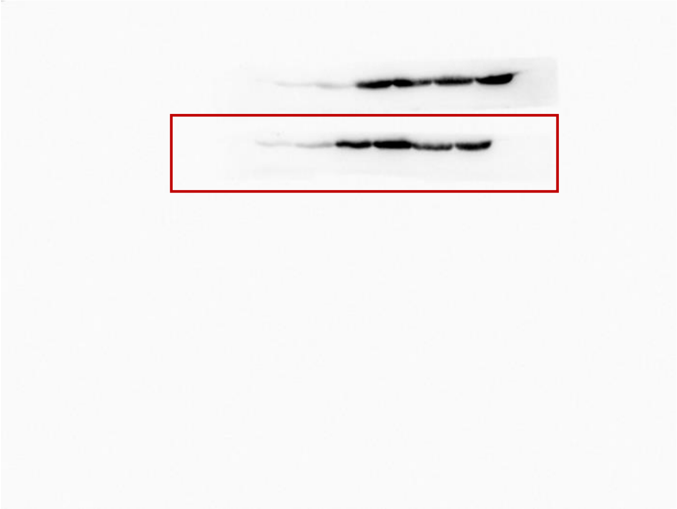

Figure 2F. α-SMA

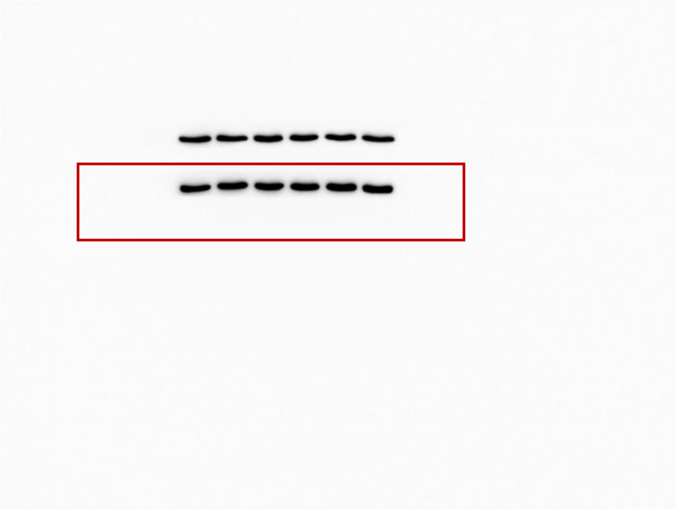

Figure 2F. β-Actin

Figure 2

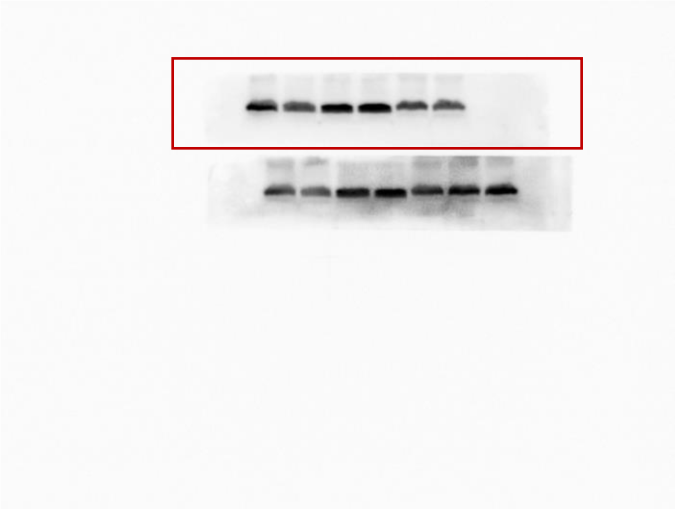

Figure 3A. p16^{INK4a}

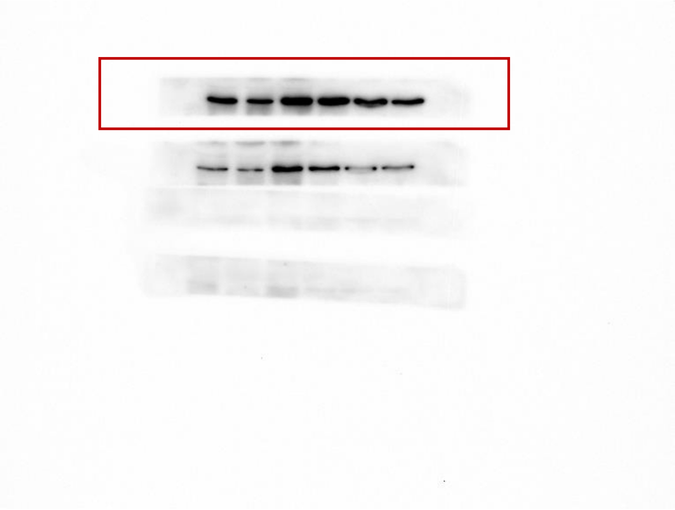

Figure 3A. p21

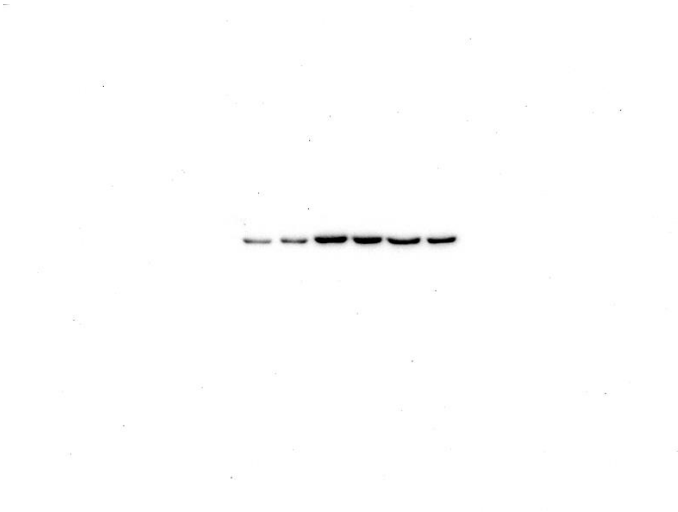

Figure 3A. p19^{ARF}

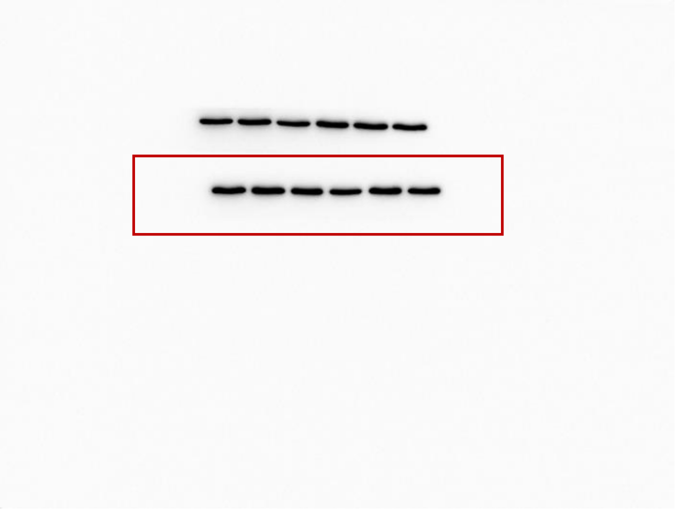

Figure 3A. β-Actin

Figure 3

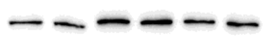

Figure 3F. active-β-catenin

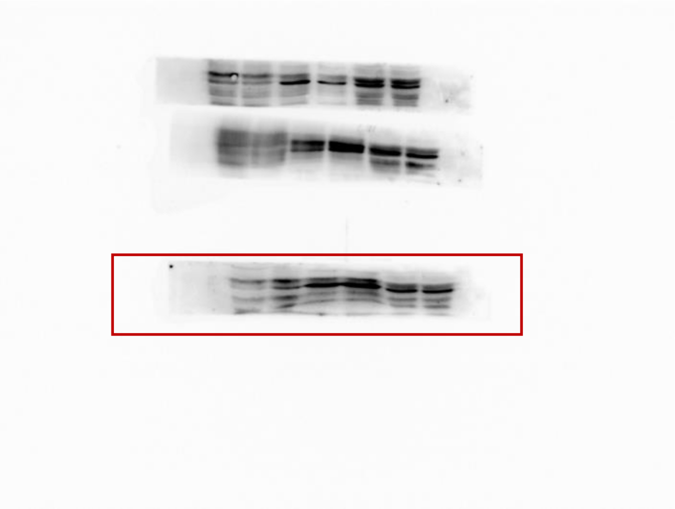

Figure 3F. MMP-7

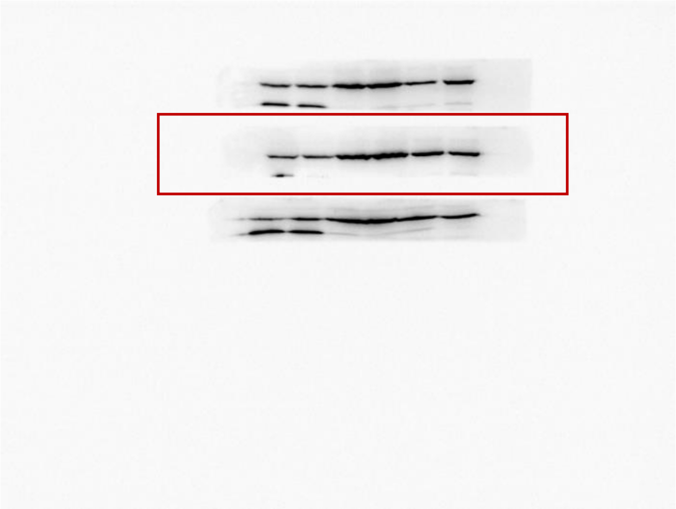

Figure 3F. Snail-1

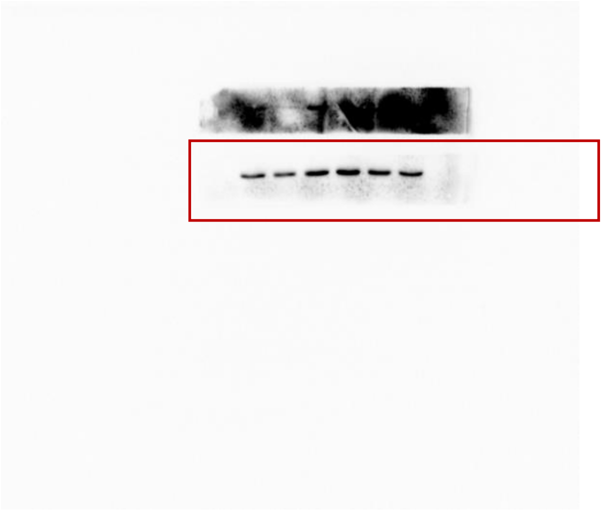

Figure 3F. PAI-1

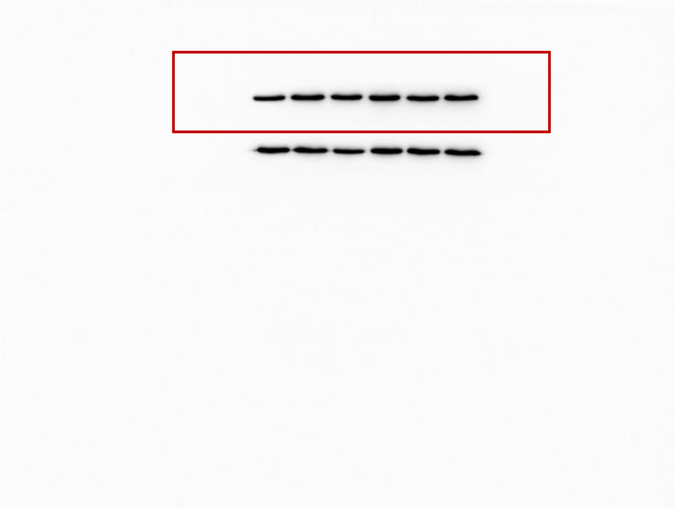

Figure 3F. β-Actin

Figure 3

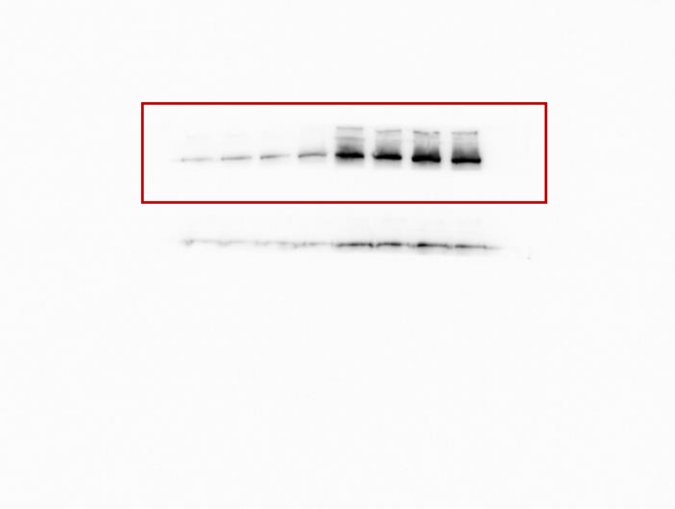

Figure 4B. BRG1

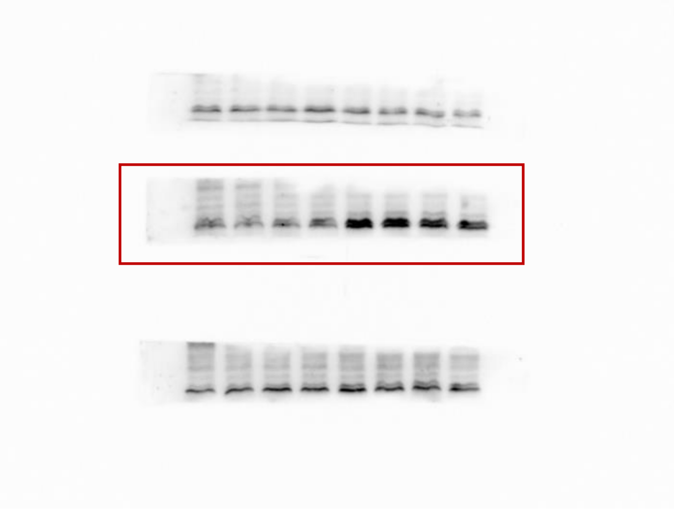

Figure 4B. p16INK4a

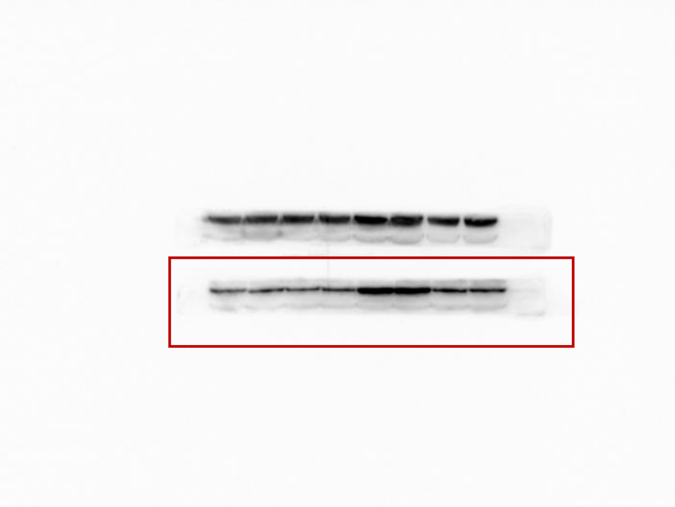

Figure 4B. p21

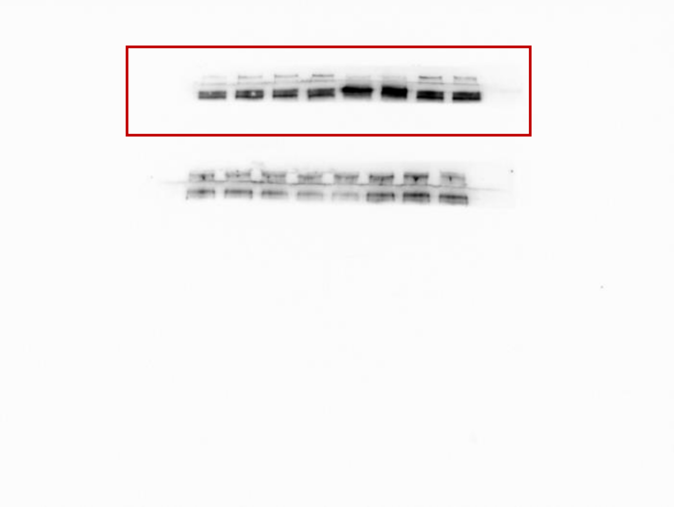

Figure 4B. Fibronectin

Figure 4

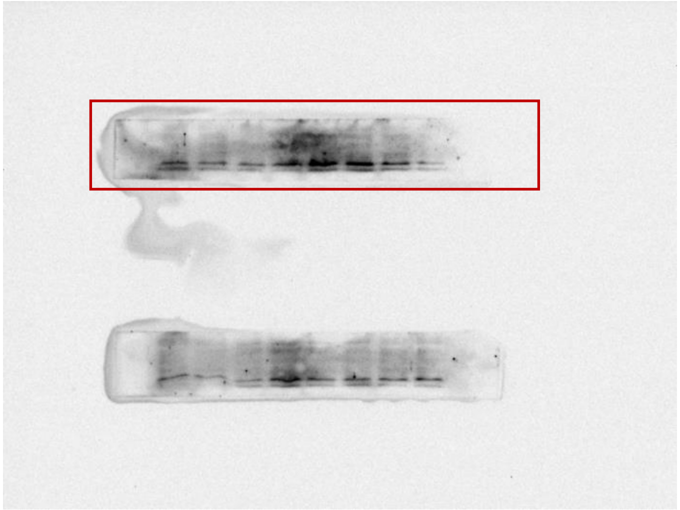

Figure 4B. Collagen I

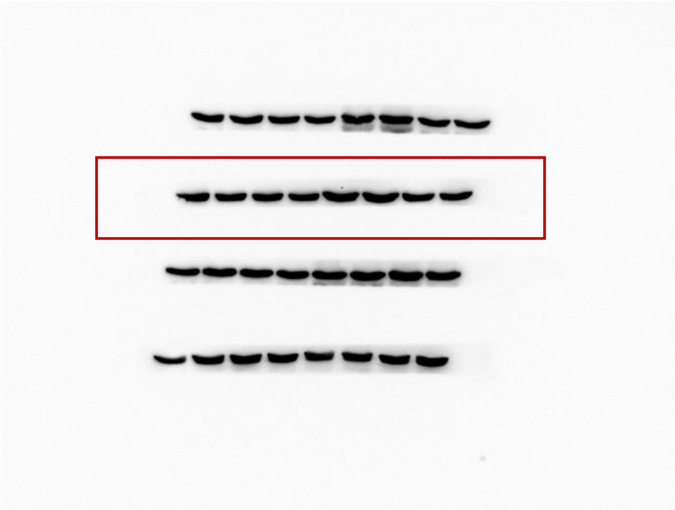

Figure 4B. α-SMA

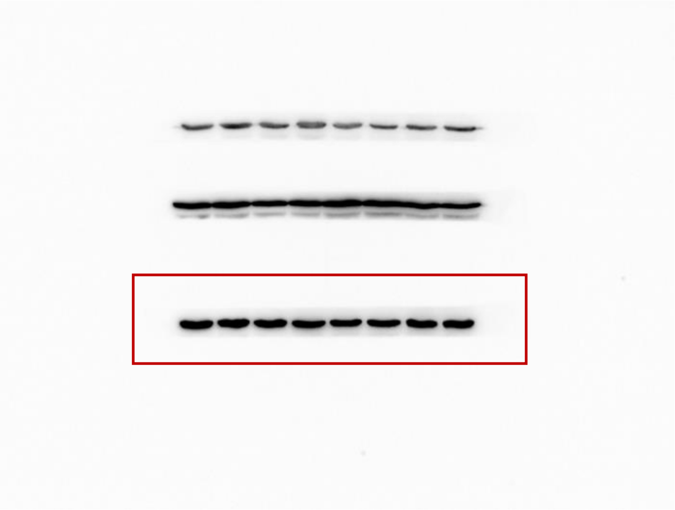

Figure 4B. β-Actin

Figure 4

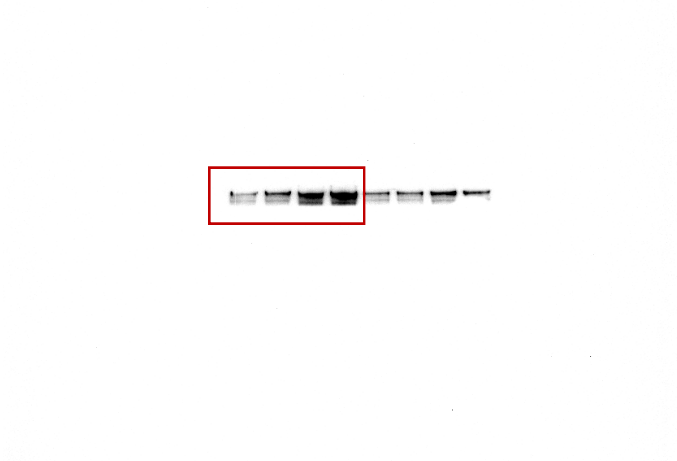

Figure 5A. BRG1

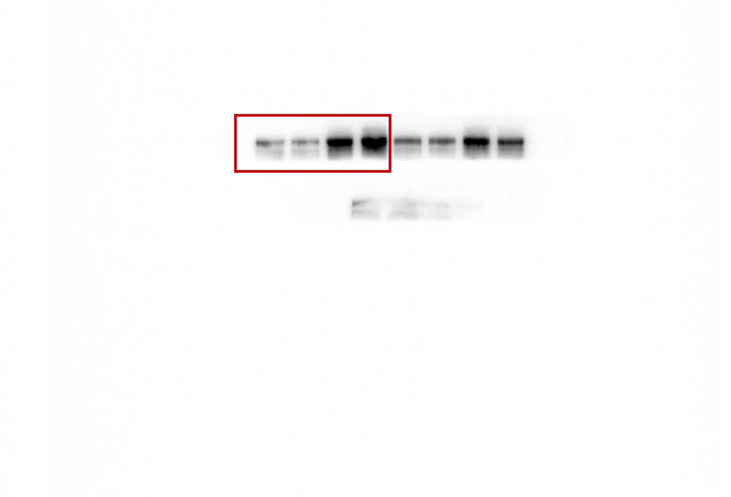

Figure 5A. Fibronectin

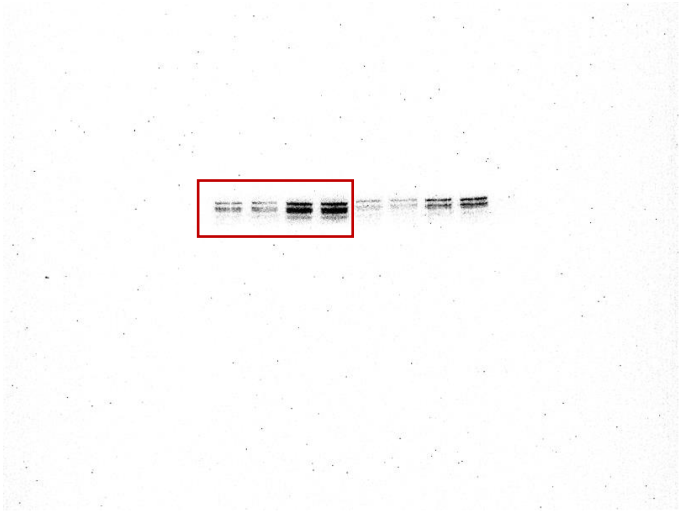

Figure 5A. Collagen I

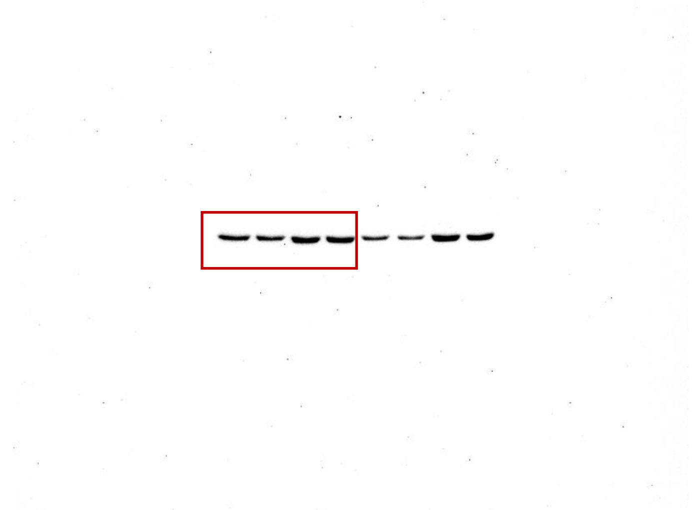

Figure 5A. α-SMA

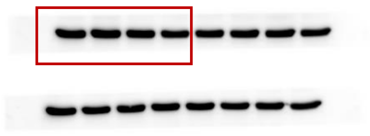

Figure 5A. β-Actin

Figure 5

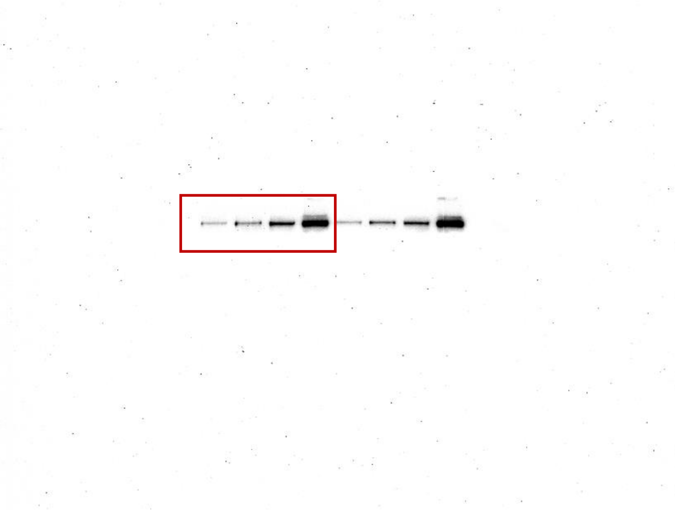

Figure 5G. BRG1

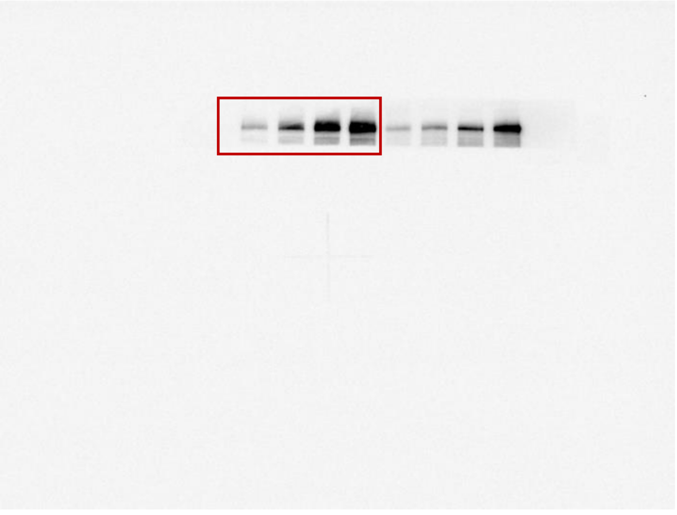

Figure 5G. Fibronectin

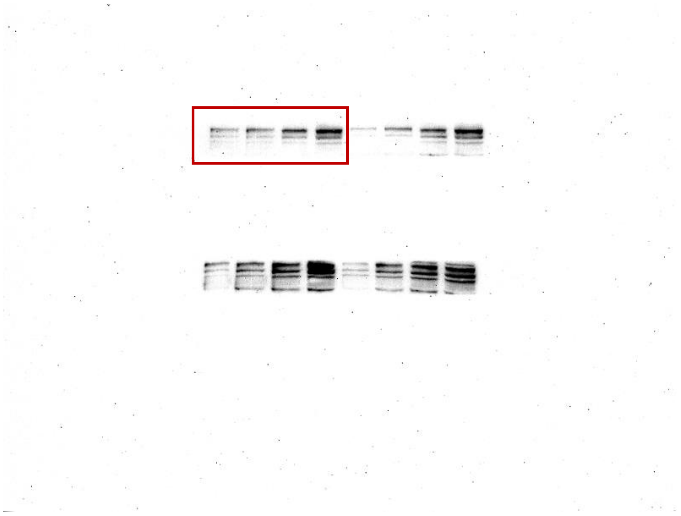

Figure 5G. Collagen I

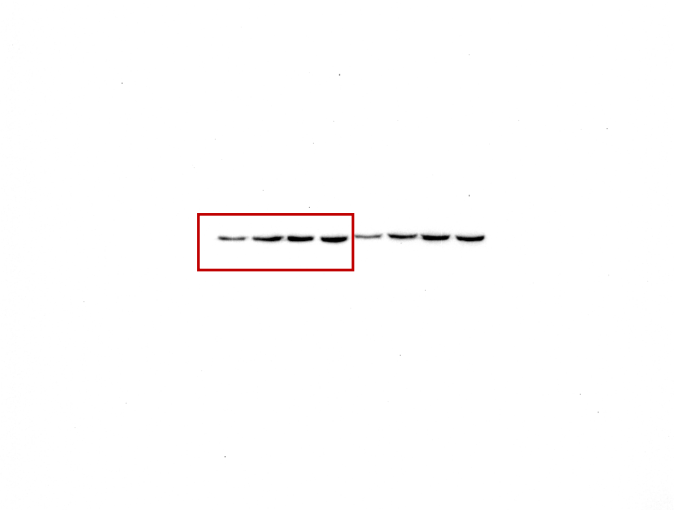

Figure 5G. α-SMA

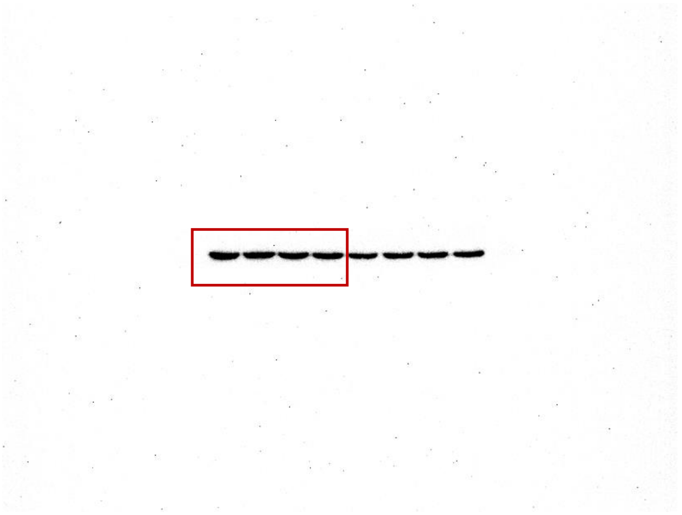

Figure 5G. β-Actin

Figure 5

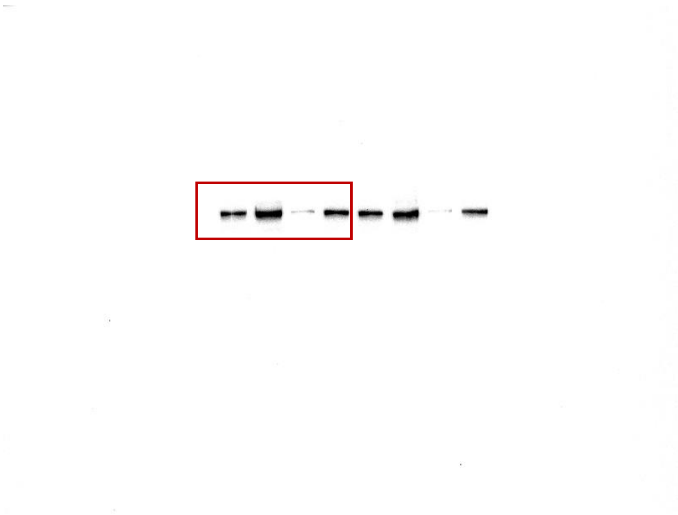

Figure 5L. BRG1

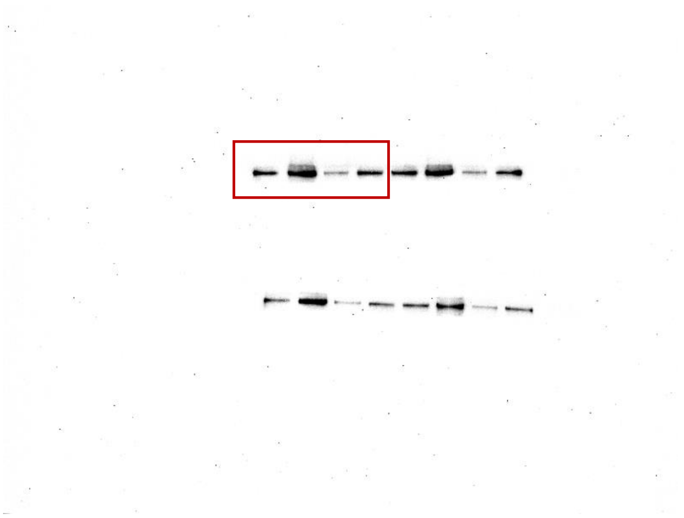

Figure 5L. Fibronectin

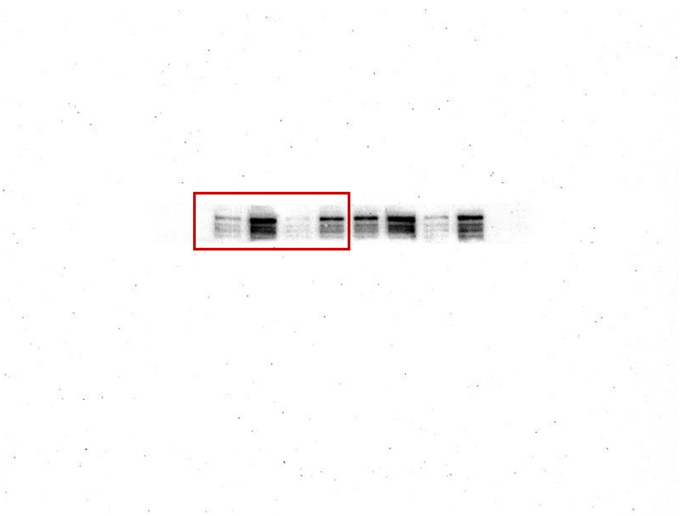

Figure 5L. Collagen I

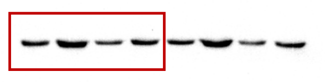

Figure 5L. α-SMA

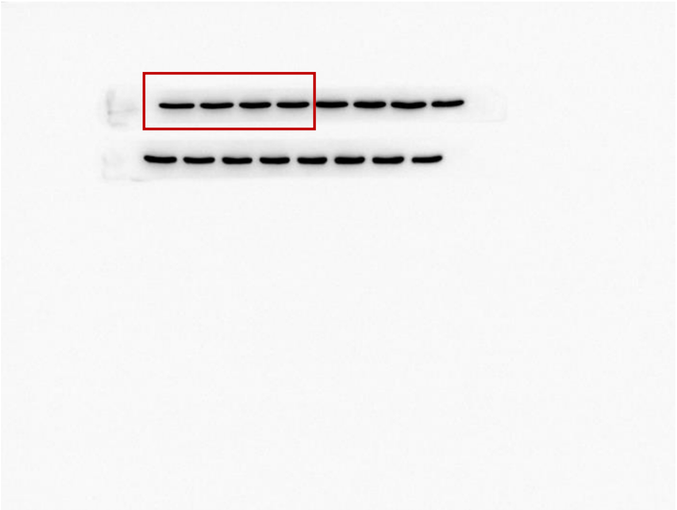

Figure 5L. β-Actin

Figure 5

Figure 6C. p16^{INK4a}

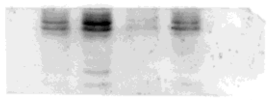

Figure 6C. p19^{ARF}

Figure 6C. p21

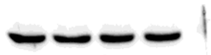

Figure 6C. β-Actin

Figure 6

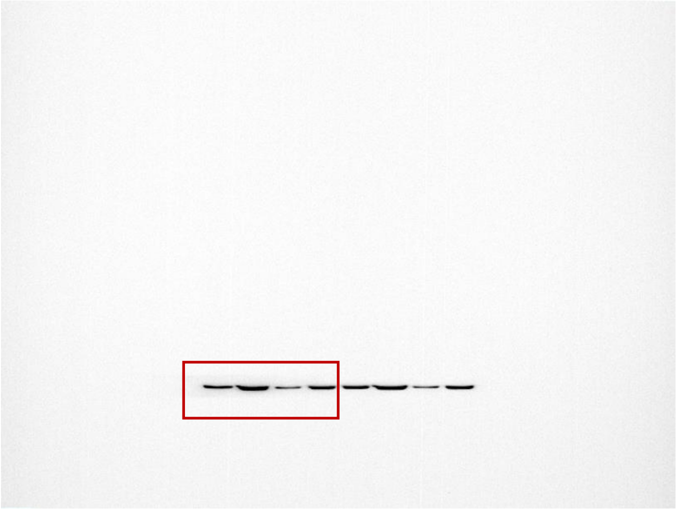

Figure 6G. p16^{INK4a}

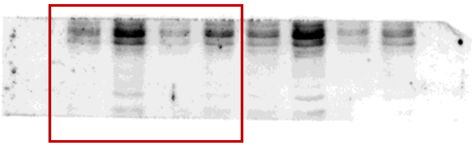

Figure 6G. p19^{ARF}

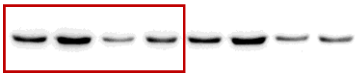

Figure 6G. p21

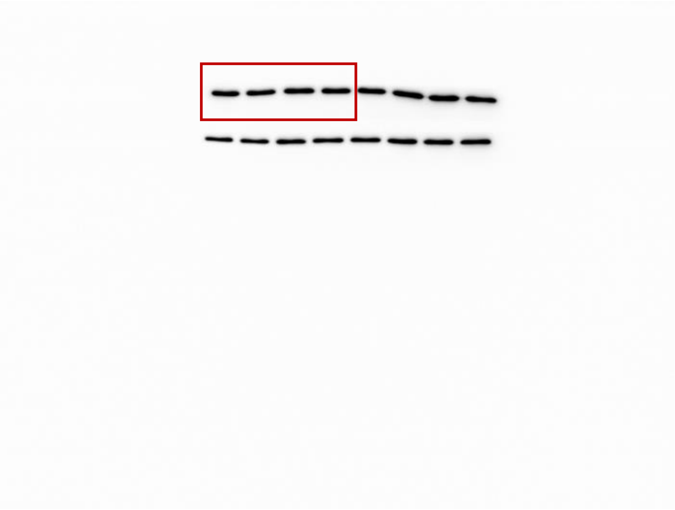

Figure 6G. β-Actin

Figure 6

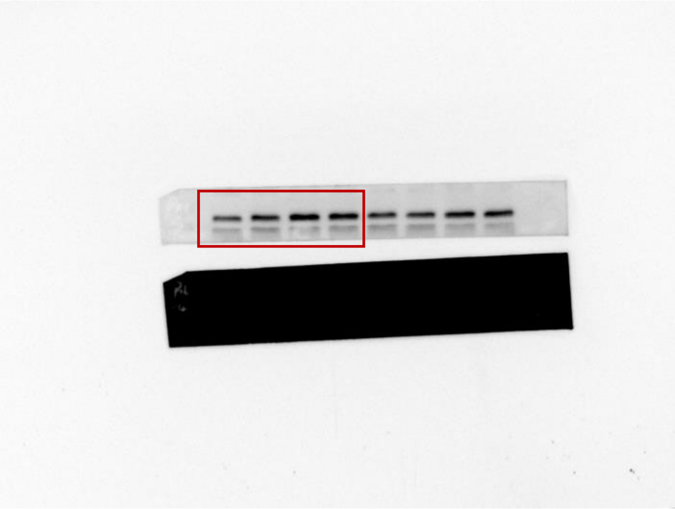

Figure 6I. p16^{INK4a}

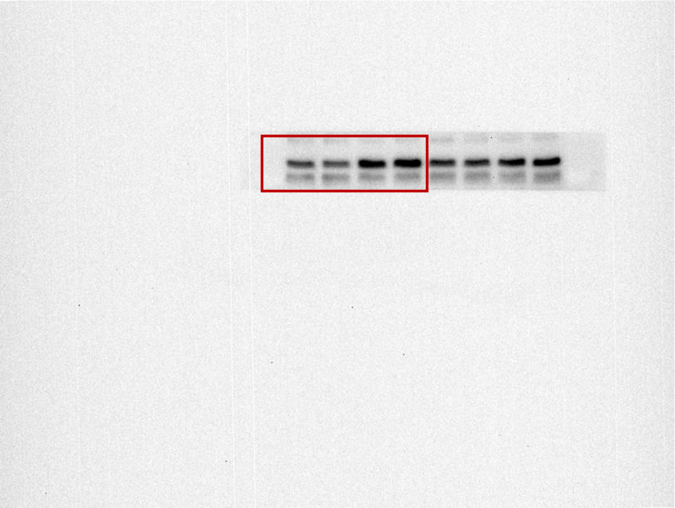

Figure 6I. p19^{ARF}

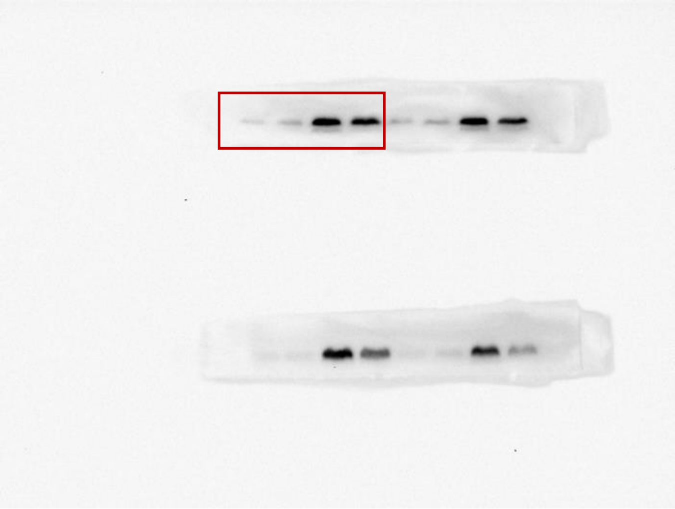

Figure 6I. p21

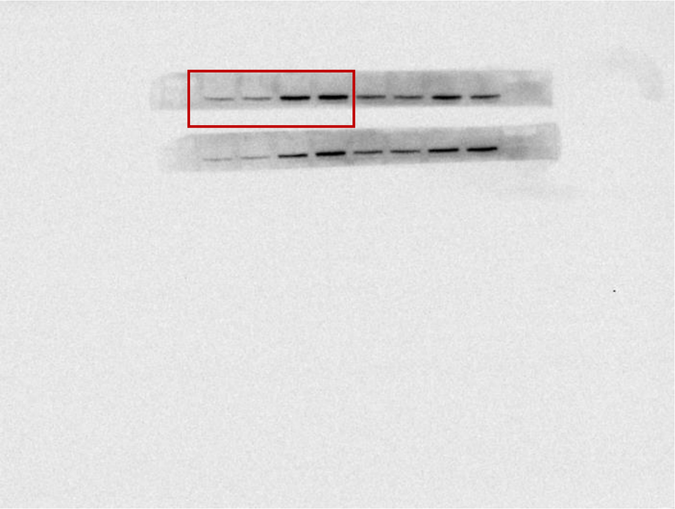

Figure 6I. TGFβ1

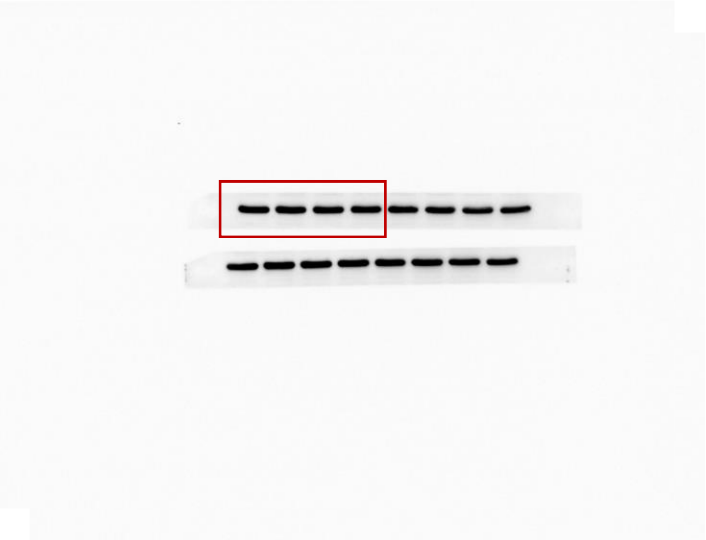

Figure 6I. β-Actin

Figure 6

Figure 7B. β-catenin

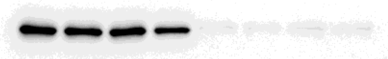

Figure 7B. β-Actin

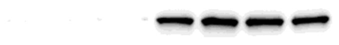

Figure 7B. Histone H3

Figure 7

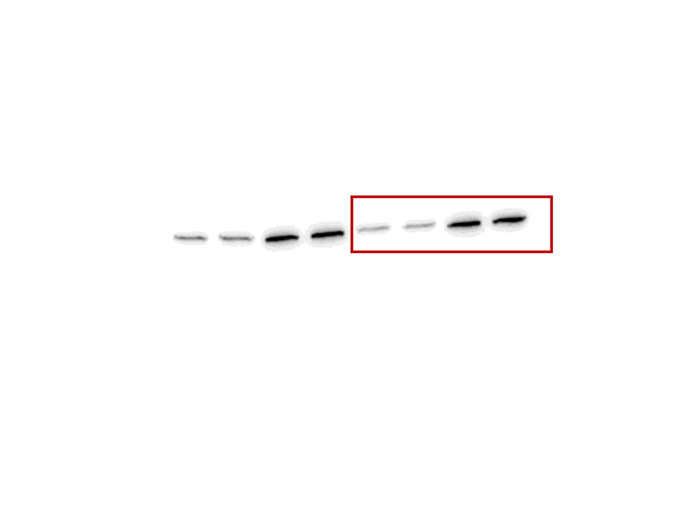

Figure 7E. active-β-catenin

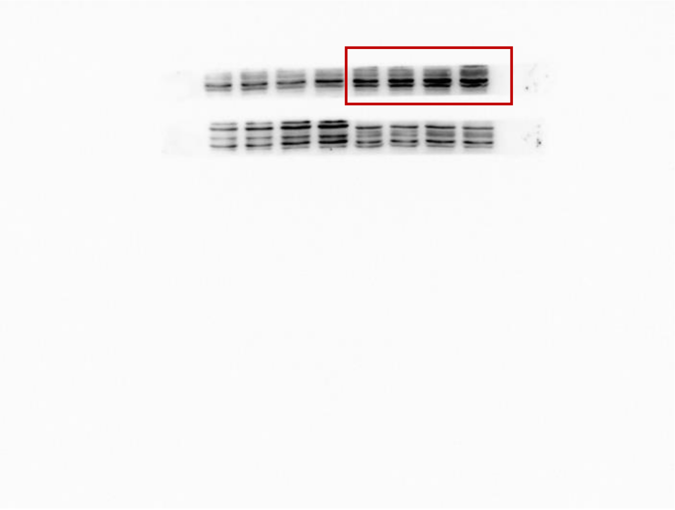

Figure 7E. MMP-7

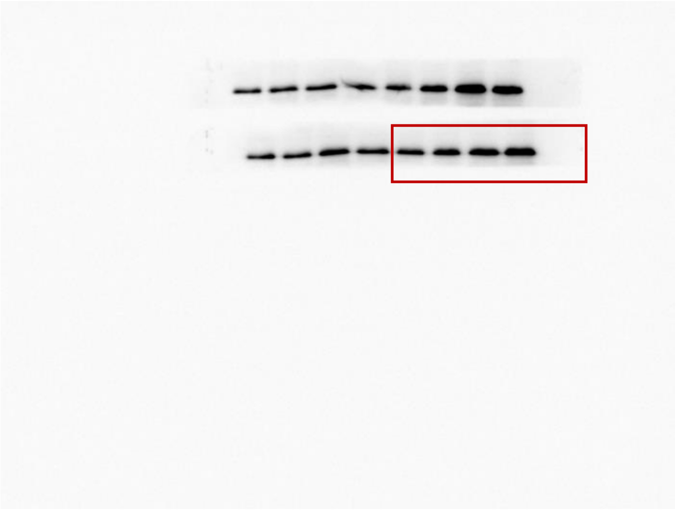

Figure 7E. Snail-1

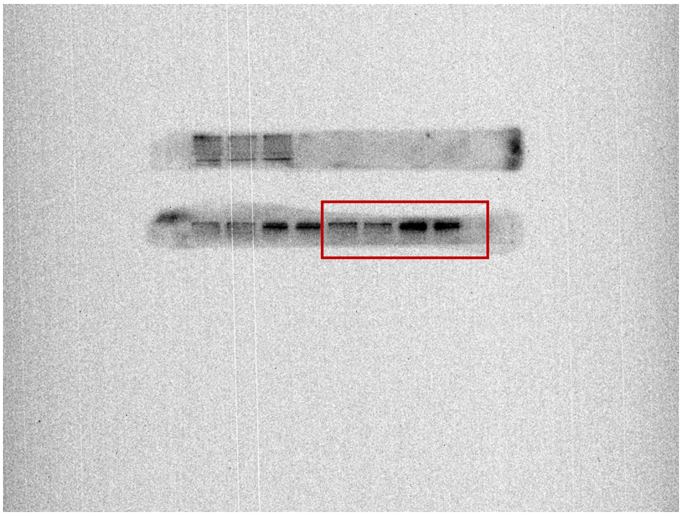

Figure 7E. PAI-1

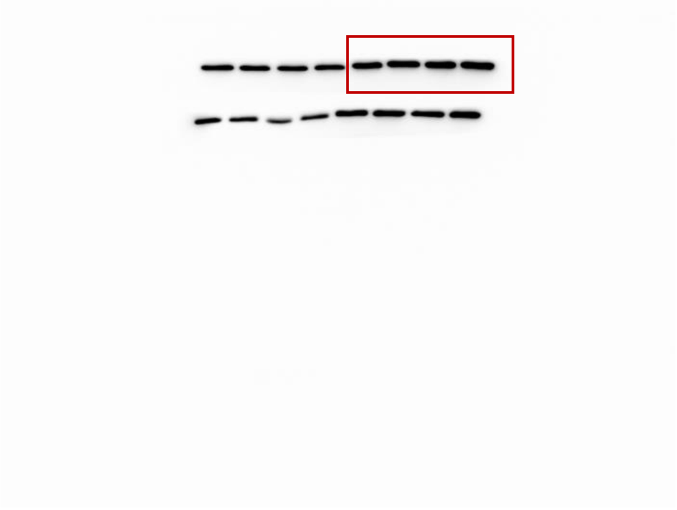

Figure 7E. β-Actin

Figure 7

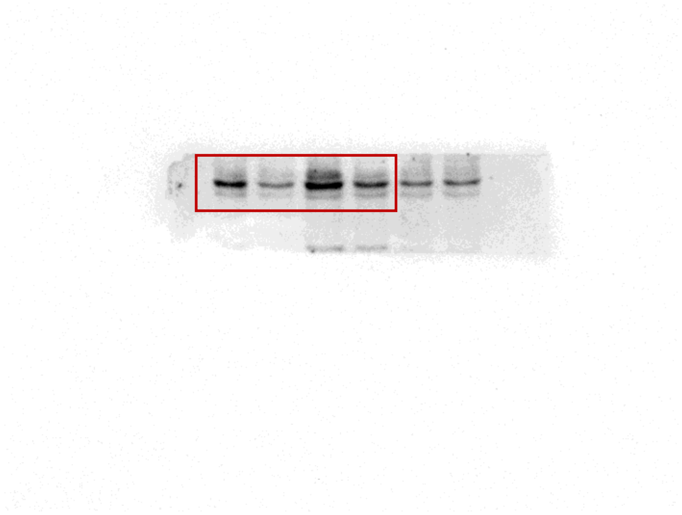

Figure 7J. p16^{INK4a}

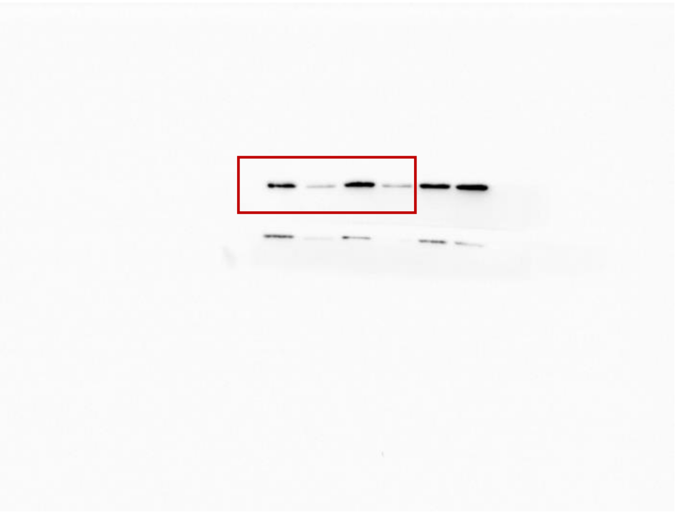

Figure 7J. p21

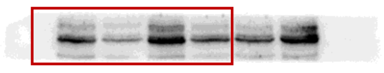

Figure 7J. p19^{ARF}

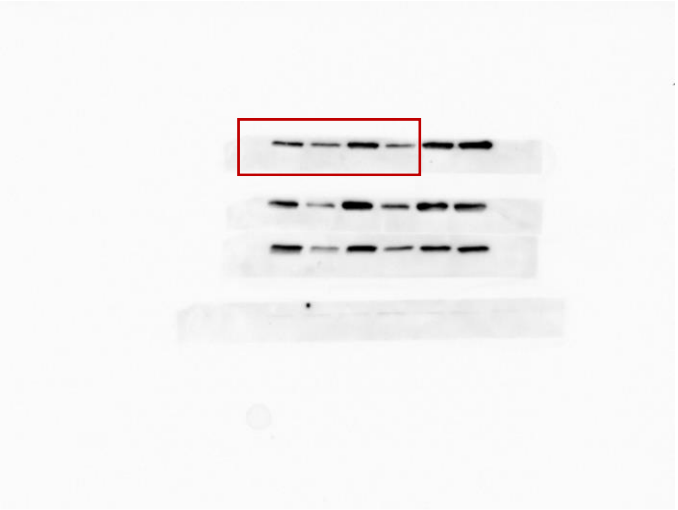

Figure 7J. TGFβ1

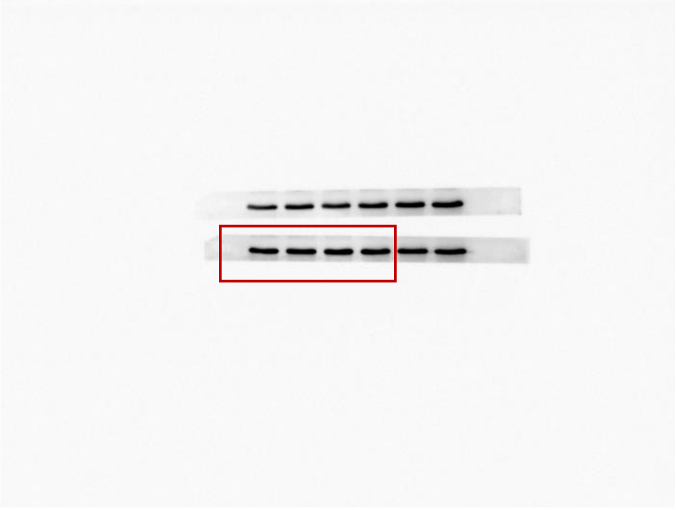

Figure 7J. β-Actin

Figure 7

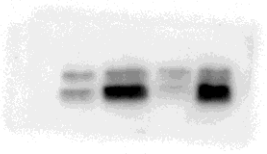

Figure 8A. LC3 A/B

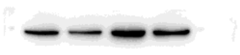

Figure 8A. SQSTM1

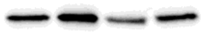

Figure 8A. Beclin-1

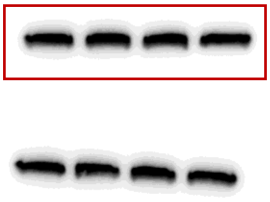

Figure 8A. β-Actin

Figure 8

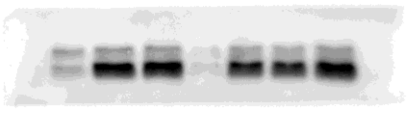

Figure 8C. LC3 A/B

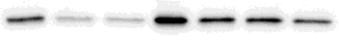

Figure 8C. SQSTM1

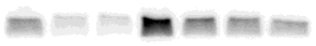

Figure 8C. p16^{INK4a}

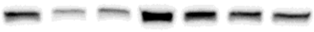

Figure 8C. p21

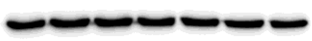

Figure 8C. β-Actin

Figure 8

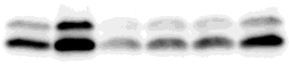

Figure 8H. LC3 A/B

Figure 8H. SQSTM1

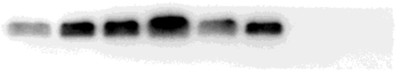

Figure 8H. $p16^{INK4a}$

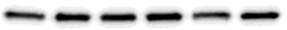

Figure 8H. p21

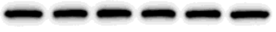

Figure 8H. β-Actin

Figure 8

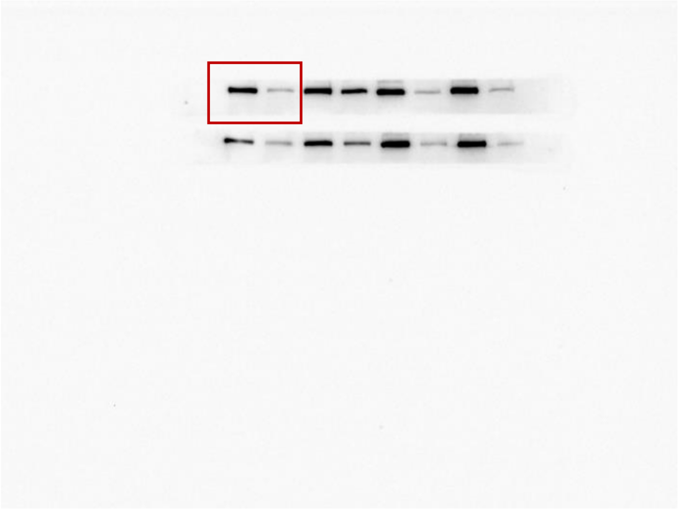

Figure 8M. BRG1

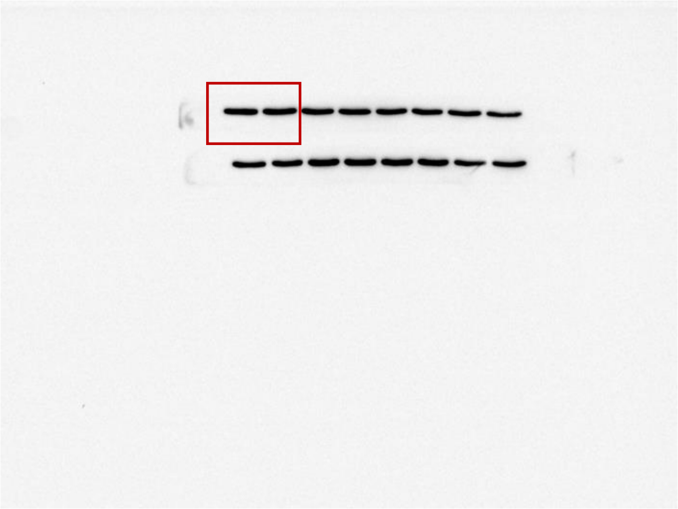

Figure 8M. β-Actin

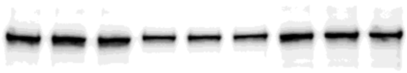

Figure 8P. BRG1

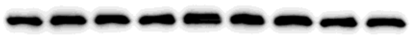

Figure 8P. β-Actin

Figure 8

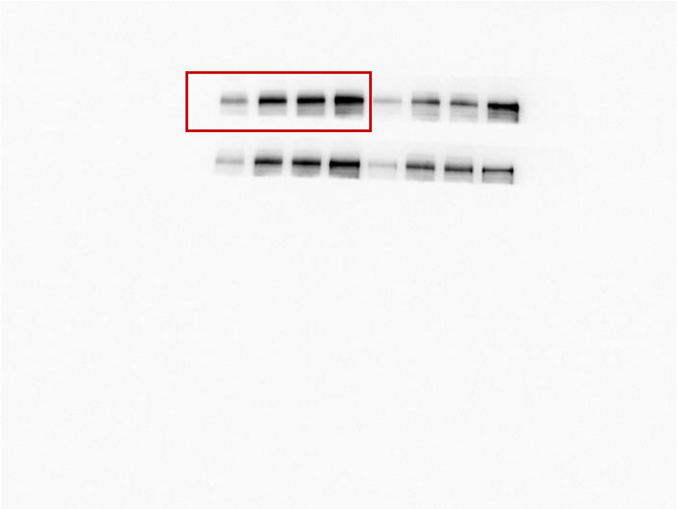

Figure 9H. Fibronectin

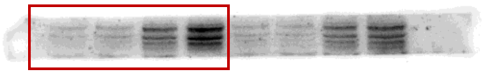

Figure 9H. Collagen I

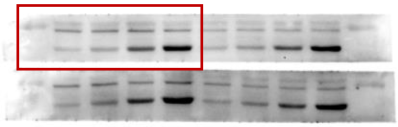

Figure 9H. α-SMA

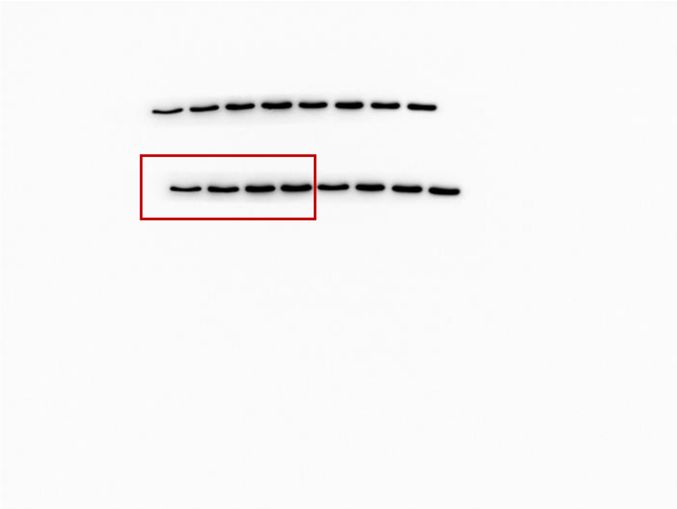

Figure 9H. PCNA

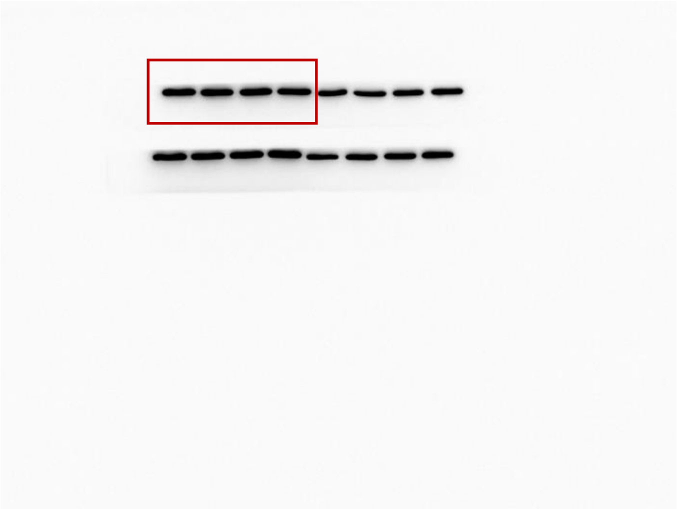

Figure 9H. β-Actin

Figure 9

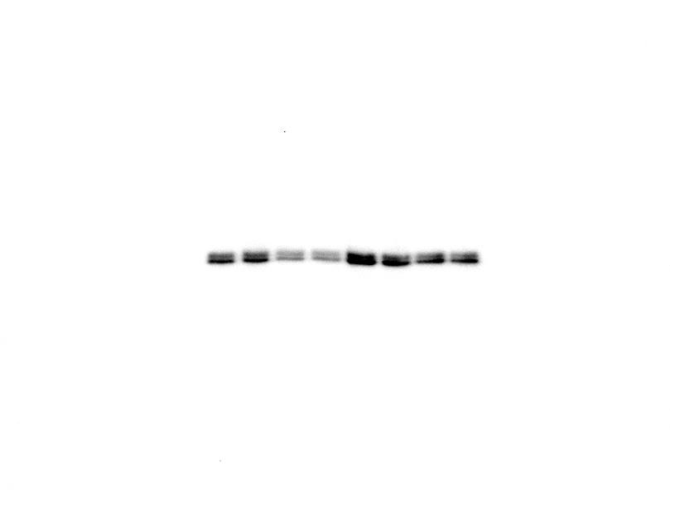

Supplementary Figure 1. BCL-xL

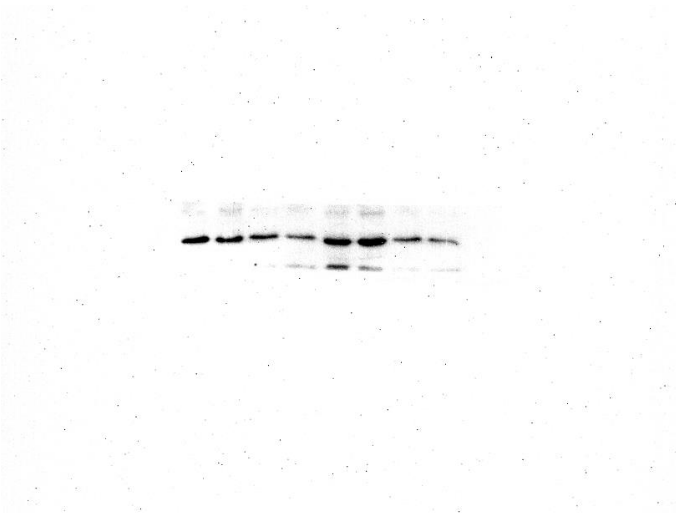

Supplementary Figure 1. BCL-2

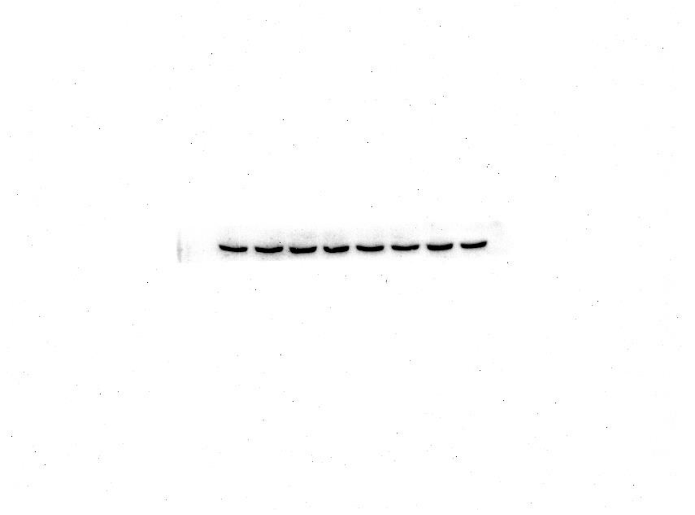

Supplementary Figure 1. β-Actin

Supplementary Figure 1

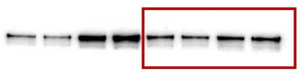

Supplementary Figure 2. BRG1

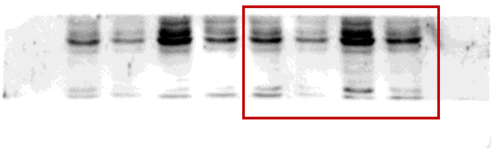

Supplementary Figure 2. p16INK4a

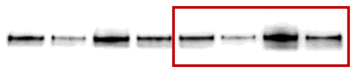

Supplementary Figure 2. Fibronectin

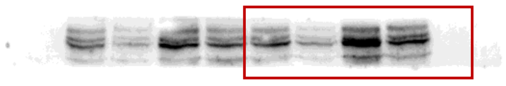

Supplementary Figure 2. Collagen I

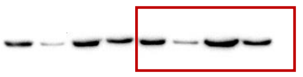

Supplementary Figure 2. α-SMA

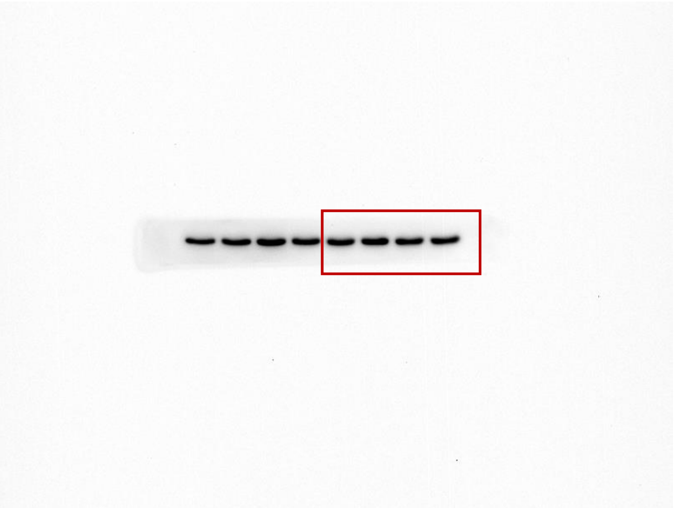

Supplementary Figure 2. β-Actin

Supplementary Figure 2

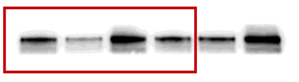

Supplementary Figure 4. Fibronectin

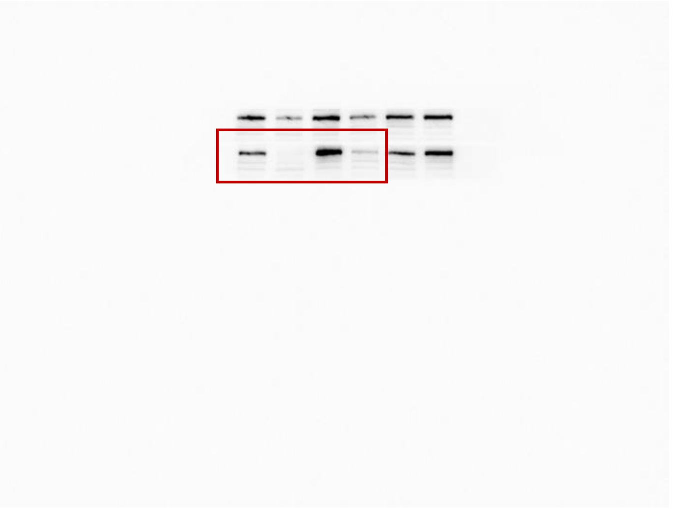

Supplementary Figure 4. Collagen I

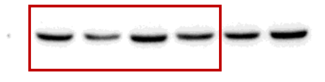

Supplementary Figure 4. α-SMA

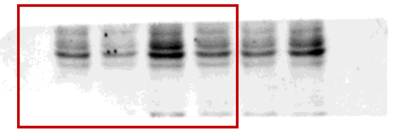

Supplementary Figure 4. PAI-1

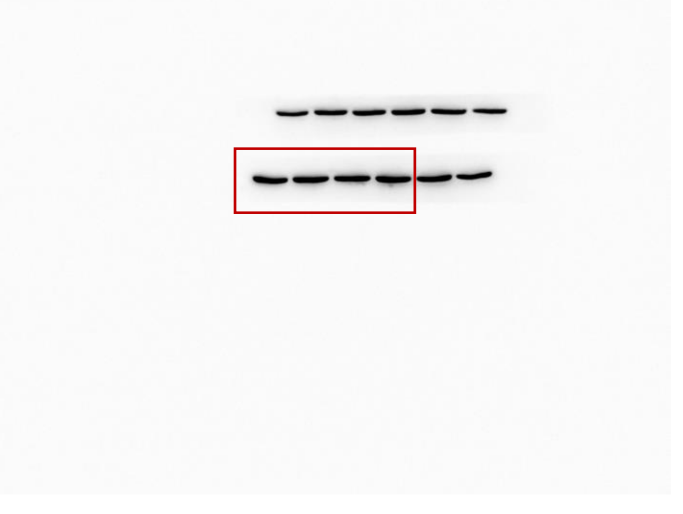

Supplementary Figure 4. β-Actin

Supplementary Figure 4

Supplementary Figure 6. Full bands of all proteins (Figure 1-9 and Supplemental Figures).

Supplementary Table 1. Primer sequences used for the qRT-PCR analysis

Gene	Forward (5'-3')	Reverse (5'-3')
β-Actin	CAGCTGAGAGGGAAATCGTG	CGTTGCCAATAGTGATGACC
Wnt1	TGACAACATCGATTTTGGTCGC	CCTCGTTGTTGTGAAGGTTCATG
Wnt2	GTGATGTGTGACAATGTGCCA	GTTGCAGTTCCAGCGATGC
Wnt2b	TTGTGTCAACGCTACCCAGA	ACCACTCCTGCTGACGAGAT
Wnt3	GGGGCGTATTCAAGTAGCTG	GTAGGGACCTCCCATTGGAT
Wnt3a	TTCTTACTTGAGGGCGGAGA	CTGTCGGGTCAAGAGAGGAG
Wnt4	CGAGGAGTGCCAATACCAGT	GTCACAGCCACACTTCTCCA
Wnt5a	CATCGACTATGGCTACCGCTT	GTATACTGTCCTACGGCCTGC
Wnt5b	GGAGATGTTCATCATTGGCGC	CTGGTGTTGGCACTCTCTGAT
Wnt6	CTCCTACAGTGTGGTTGTCAGG	GCGCATCCATAAAGAGTCTTGA
Wnt7a	GACAAATACAACGAGGCCGT	GGCTGTCTTATTGCAGGCTC
Wnt7b	CTTCACCTATGCCATCACGG	TGGTTGTAGTAGCCTTGCTTCT
Wnt8a	AAAGGGAAGGATGCCAGAGC	TTTCCCATCTGCCGGAAGTC
Wnt8b	CTGCACAACAATGAGGCTGG	CGCCGCATGATACTTCTCCT
Wnt9a	TCGTGGGTGTGAAGGTGATAA	TCCAGTGGCTTCATTGGTAGT
Wnt9b	GGGTGTGTGTGGTGACAATC	TCCAACAGGTACGAACAGCA
Wnt10a	CAGATCGCCATCCATGAGTG	ACCGCAAGCCTTCAGTTTACC
Wnt10b	ATCGCCGTTCACGAGTGTC	GGAAACCGCGCTTGAGGAT
Wnt11	ATGCGTCTACACAACAGTGAAG	GTAGCGGGTCTTGAGGTCAG
Wnt16	CCCTCTTTGGCTATGAGCTG	TACTGGACATCATCCGAGCA
FZD1	GCCAGCCATCAAAACTATAACC	GTGCCAATGAACAGATAAACGA
FZD2	CGTATACCTGTTCATCGGTACA	GAAGGCCTGCTCATAGAAGTAG
FZD3	CTGGATTGTTCTCGGGATTTTC	AAATTCAAATCCACAAGTCGGG
FZD4	TGGAAAGGCTAATGGTCAAGAT	CCCACGAGCAAAGACATAAAAA
FZD5	TCTTCCTGTGCTCTATGTACAC	TAATCCATACACAGAACCTCGG
FZD6	CGGTCTTCTGGGTTGGAAGC	CAGGACTCTTGCAGCACTCG
FZD7	CAGGACTCTTGCAGCACTCG	CTCATAAAAGTAGCAGGCCAAC

FZD8	CATATTCCTCTCCGCCTGTTAC	CAGTGGTTTCATAGCGAACATG
FZD9	TGGTTTTGACTCTAACCTGGTT	CAGTACCAGGTAGCAAGATAGG
FZD10	GAGAAGCTCATGGTACGCATAG	CCATGTTGAGGCGTTCATAAAA
LRP5	CACACCATGCGAGGAGGACAAC	CGAGCCAGCAGCAGCACTTC
LRP6	TGCGAAGACCACAGCGATGAAC	TGACCATTGGCACAGCGGAAC
GSK3b	CAACGCCACAGCAGCCTCAG	CAGGTGGAGTTGGAAGCTGATGC
DVL1	ATGGCGGAGACCAAAATCATC	AACTTGGCATTGTCATCGAAGA
DVL2	TGTCGTCAGATACCCCACAG	CTGGATACATTAGGGTGGAAGGA
Axin1	CTCCAAGCAGAGGACAAAATCA	GGATGGGTTCCCCACAGAAATA
